# Protein Target Highlights in CASP16: Insights From the Structure Providers

**DOI:** 10.1002/prot.70025

**Published:** 2025-10-09

**Authors:** Leila T. Alexander, Océane M. Follonier, Andriy Kryshtafovych, Kim Abesamis, Sabrina Bibi‐Triki, Henry G. Box, Cécile Breyton, Françoise Bringel, Loic Carrique, Alessio d'Acapito, Gang Dong, Rebecca DuBois, Deborah Fass, Juliana Martinez Fiesco, Daniel R. Fox, Jonathan M. Grimes, Rhys Grinter, Matthew Jenkins, Roman Kamyshinsky, Jeremy R. Keown, Gerald Lackner, Michael Lammers, Shiheng Liu, Andrew L. Lovering, Tomas Malinauskas, Benoît Masquida, Gottfried J. Palm, Christian Siebold, Tiantian Su, Ping Zhang, Z. Hong Zhou, Krzysztof Fidelis, Maya Topf, John Moult, Torsten Schwede

**Affiliations:** ^1^ Biozentrum University of Basel Basel Switzerland; ^2^ Computational Structural Biology SIB Swiss Institute of Bioinformatics Basel Switzerland; ^3^ Transplantation & Clinical Virology, Department of Biomedicine University of Basel Basel Switzerland; ^4^ Genome Center University of California Davis California USA; ^5^ Max Perutz Labs, Vienna Biocenter Medical University of Vienna Vienna Austria; ^6^ Vienna Biocenter PhD Program A Doctoral School of the University of Vienna and the Medical University of Vienna Vienna Austria; ^7^ UMR 7156, GMGM Université de Strasbourg – CNRS Strasbourg France; ^8^ Department of Biosciences University of Birmingham UK; ^9^ University Grenoble Alpes, CNRS, CEA, IBS Grenoble France; ^10^ Division of Structural Biology, Centre for Human Genetics University of Oxford Oxford UK; ^11^ Department of Biomolecular Engineering University of California Santa Cruz California USA; ^12^ Department of Chemical and Structural Biology Weizmann Institute of Science Rehovot Israel; ^13^ Kinase Complexes Section, Center for Structural Biology, Center for Cancer Research National Cancer Institute Frederick Maryland USA; ^14^ Department of Microbiology, Biomedicine Discovery Institute Monash University Melbourne Victoria Australia; ^15^ Department of Biochemistry and Pharmacology, Bio21 Molecular Science and Biotechnology Institute The University of Melbourne Melbourne Victoria Australia; ^16^ Centre for Electron Microscopy of Membrane Proteins Monash Institute of Pharmaceutical Sciences Melbourne Victoria Australia; ^17^ Department of Chemical Research Support Weizmann Institute of Science Rehovot Israel; ^18^ School of Life Sciences University of Warwick Coventry UK; ^19^ Chair of Biochemistry of Microorganisms University of Bayreuth Kulmbach Germany; ^20^ Institute of Biochemistry University of Greifswald Greifswald Germany; ^21^ Department of Microbiology, Immunology, and Molecular Genetics University of California Los Angeles California USA; ^22^ California NanoSystems Institute University of California Los Angeles California USA; ^23^ Leibniz Institute of Virology and Centre for Structural Systems Biology Deutsches Elektronen‐Synchrotron Hamburg Germany; ^24^ Institute for Molecular Virology and Tumorvirology University Medical Center Hamburg‐Eppendorf Germany; ^25^ Department of Cell Biology and Molecular Genetics, Institute for Bioscience and Biotechnology Research University of Maryland Rockville Maryland USA

**Keywords:** CASP, cryo‐EM, protein structure prediction, X‐ray crystallography

## Abstract

This article presents an in‐depth analysis of selected CASP16 targets, with a focus on their biological and functional significance. The authors highlight the most relevant features of the target proteins and discuss how well these were reproduced in the submitted predictions. While the overall performance of structure prediction methods remains impressive, challenges persist, particularly in modeling rare structural motifs, flexible regions, small molecule interactions, posttranslational modifications, and biologically important interfaces. Addressing these limitations can strengthen the role of structure prediction in complementing experimental efforts and advancing both basic research and biomedical applications.

**Abbreviations**
ASU: asymmetric unit
BMP: bone morphogenetic proteins
cAMP: cyclic adenosine monophosphate
CASP: community‐wide experiment on the Critical Assessment of Techniques for Protein Structure Prediction
CCD: central conserved domain
CDR: complementarity‐determining region
COR: C‐terminal of Roc
cryo‐EM: cryo‐electron microscopy
DG: dystroglycan
DGC: dystrophin‐glycoprotein complex
FABP: fatty acid binding protein
Fgd: F420‐dependent glucose‐6‐phosphate dehydrogenase
FPC: flagellar pocket collar
GDF5: growth differentiation factor 5
GDT: global distance test
ICS score: interface contact similarity score
LDDT: local distance difference test
LGA: local group alignment
LRRK2: leucine‐rich repeat kinase 2
LTFs: lateral tail fibers
MSA: multiple sequence alignment
Mtase: methyltransferase domain
NbE11: nanobody E11
nsNSV: non‐segmented negative‐sense RNA viruses
PD: Parkinson's disease
PDB: Protein Data Bank
PRNTase: polyribonucleotidyltransferase domain
RdRp: RNA‐dependent RNA polymerase domain
RMSD: root mean square deviation
Roc: Ras of complex protein
RSV: respiratory syncytial virus
SAD: single‐wavelength anomalous diffraction
SG: sarcoglycan
TBDT: TonB‐dependent transporter
TM: transmembrane
TWSG1: twisted gastrulation 1

## 1 Introduction

The success of CASP would not be possible without the invaluable contributions of experimental structural biologists, who share their work‐in‐progress with the CASP organization. In the latest round of CASP (Reference to the “Critical assessment of methods of protein structure prediction (CASP)—Round XVI” paper in this issue), 56 structure determination groups from 15 countries suggested 103 structures as prediction targets, with the largest contributions coming from the United States (23 groups providing 46 targets) and the United Kingdom (8 groups, 16 targets). The CASP16 target set is very diverse and includes single‐sequence protein molecules, protein–protein complexes, RNA and DNA molecules (monomers and multimers), RNA–protein complexes, receptor–ligand complexes and three special interest targets: two semi‐disordered protein systems consisting of two rigid domains joined by flexible linkers, and an RNA molecule with solvent shell. 50 structures were solved by X‐ray crystallography, 49 by cryo‐EM and 4 by NMR. The CASP organizers, who are co‐authors of this article, are grateful to the experimentalists that provided targets for CASP16, thereby contributing to the development of more accurate biomolecular structure prediction methods.

This manuscript is the eighth in a series of CASP target highlight papers [1, 2, 3, 4, 5, 6, 7]. It presents accounts by the authors of the selected protein targets, representing the respiratory syncytial virus (RSV) glycoprotein G in complex with human antibodies (H1222, H1223, and H1225), bornavirus polymerase complex (H1220 and T1220S1), bacteriophage T5 lateral tail fiber pb1 (T1257), the DcmC protein from the ***dcm*** operon of ***Methylobacterium*** *extroquens* (T1246), F_420_‐dependent glucose‐6‐phosphate dehydrogenase (T1278), a cyclic nucleotide‐binding protein from gram‐negative *Bdellovibrio bacteriovorus* (T1298), filaments of human α‐defensin 6 (HD6) (T1219), human twisted gastrulation 1 (T1201), the hemoglobin‐NbE11 nanobody complex (H1204), the nanobody Nb48 bound to the coiled‐coil domain of BILBO1 (H1244), the LRRK2:14‐3‐3_2_ complex (H1258), and the rabbit dystrophin–glycoprotein complex DGC (H0272, H1272, and H2272).

Two sister articles in this issue provide experimentalists' reports on the nucleic‐acid‐containing targets and protein‐ligand targets from pharmaceutical discovery projects. The results of the comprehensive numerical evaluation of CASP16 models are available on the Prediction Center website (http://www.predictioncenter.org). The detailed assessment of the models by the assessors is provided elsewhere in this issue.

## 2 Results

### 2.1 Structures of the RSV Glycoprotein G Central Conserved Domain (CCD) Bound to Monoclonal Antibodies (CASP: H1222, H1223, and H1225, PDB: 9CQD, 9CQB, and 9CQA). Provided by Rebecca DuBois

RSV is the top cause of severe respiratory disease in infants worldwide. The RSV glycoprotein G is responsible for virus attachment to airway epithelial cells and modulation of host immune responses. The extracellular region of RSV G contains two highly glycosylated and variable mucin‐like domains that flank a ~40 amino acid CCD. Antibodies targeting the CCD have been shown to neutralize virus infection in vitro and provide protection from RSV infection and disease in vivo.

**FIGURE 1:**
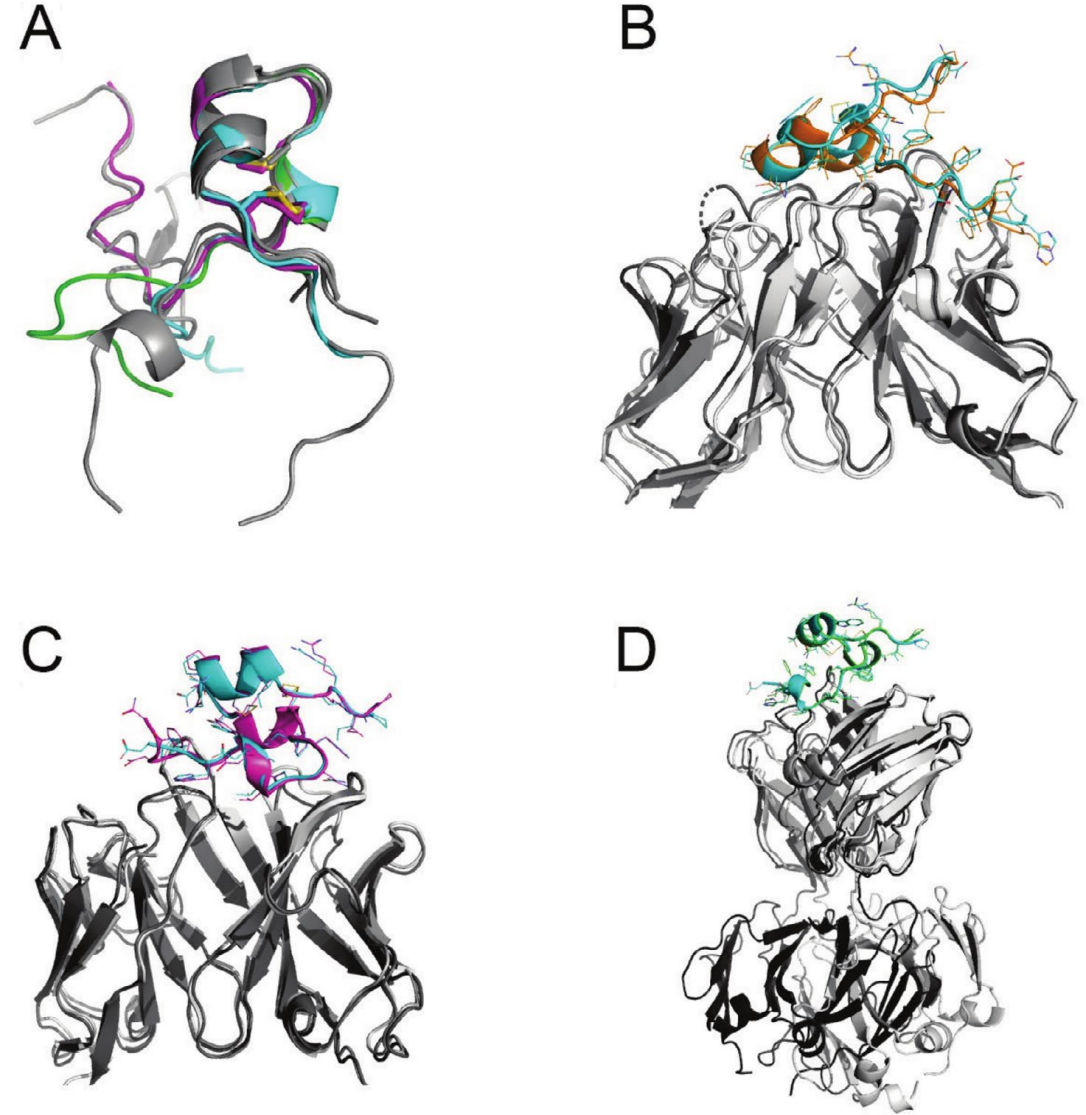
Comparison of RSV G CCD–antibody targets with CASP16 predictions. (A) Alignment of the RSV G CCD from published crystal structures (gray, PDB codes 5WNA, 5WNB, 6UVO, 6BLH, 6BLI) and from crystal structures of CASP16 targets H1222 (cyan), H1223 (magenta), and H1225 (green). (B) Alignment of the crystal structure of the RSV G CCD—antibody Fab 2B11 complex (dark gray and cyan) with the CASP16 prediction for H1222 with the top ICS (light gray and orange). (C) Alignment of the crystal structure of the RSV G CCD–antibody Fab 1G8 complex (dark gray and cyan) with the CASP16 prediction for H1223 with the top ICS (light gray and magenta). (D) Alignment of the crystal structure of the RSV G CCD–antibody Fab 1G8 complex (dark gray and cyan) with the CASP16 prediction for H1223 with a top ICS (light gray and green). Note the alignment of the CCDs and the Fab variable domains, but the poor alignment in the Fab constant domains.

Before CASP16, there were five published structures of monoclonal antibodies bound to the RSV G CCD [8, 9, 10]. Those structures revealed that the CCD antigen can adopt multiple conformations when bound by monoclonal antibodies (Figure 1A). Nevertheless, the CCD's cysteine loop, which has two disulfide bonds in a 1–4 and 2–3 connection, was structurally similar in all the structures (Figure 1A).

We solved the crystal structures of three additional monoclonal antibody Fab fragments bound to the RSV G CCD and submitted them as targets for CASP16: H1222, H1223, and H1225, with resolutions of 3.10 Å, 2.50 Å, and 1.74 Å, respectively (Table 1). We have deposited these structures in the PDB and described them in a recent publication, also reporting their binding affinities (Table 1) [11]. These structures reveal the CCD in new conformations when bound to these antibodies (Figure 1A).

**TABLE 1:** Summary of RSV G CCD–antibody Fab complex structures submitted to CASP16 with corresponding prediction assessment metrics.

Structure	CASP16 Target	Resolution	PDB ID code	Binding affinity (*K*_D_)	Interface area	Top ICS	IPS^a^	QS‐best^b^	RMSD (Å^2^)	Groups
RSV G CCD – Fab 2B11	H1222	3.10 Å	9CQD	3.2 pM	1130 Å^2^	0.870, 0.822	0.872, 0.858	0.935, 0.902	1.387, 1.554	Xgroup, DeepFold
RSV G CCD – Fab 1G8	H1223	2.50 Å	9CQB	5.3 pM	1041 Å^2^	0.874, 0.871	0.853, 0.871	0.922, 0.924	1.098, 5.855	PEZYFoldings, kozakovvajda
RSV G CCD – Fab 1G1	H1225	1.74 Å	9CQA	4.5 nM	1084 Å^2^	0.625, 0.623	0.674, 0.657	0.604, 0.611	5.758, 4.733	CSSB_experimental, MultiFOLD2

*Note*: The table includes experimental structure details, binding affinities, interface areas, top‐performing groups, and the respective values for each predicted target.

Abbreviations: ICS, interface contact score; IPS, interface packing score; QS, quality score; RMSD, root mean square deviation.

^a^ IPSs associated with the top ICSs.

^b^ QS‐bests associated with the top ICSs.

Predicting the structures of antibody–antigen complexes has been historically challenging, primarily due to the high sequence variability and structural flexibility of antibody complementarity loops that interact with the antigen. We presumed that predicting the complex with a flexible antigen would pose additional challenges. In CASP16, two of our three RSV G CCD—antibody targets were predicted with impressive accuracies. For target H1222, several groups correctly modeled the CCD antigen Cα backbone conformation and its interactions with the antibody, with the top interface contact score (ICS) of 0.870, consistent with the correct prediction of sidechain orientations and interactions at the interface (Table 1, Figure 1B). Similarly, in target H1223, several groups accurately predicted the correct conformation of the CCD antigen and its interactions with the antibody, with the top ICS of 0.874 (Table 1, Figure 1C). Interestingly, some models accurately depicted the interface but had a poor overall Cα RMSD due to differences in the overall antibody structure (Target H1223, ICS of 0.871, RMSD of 5.855 Å [2]) (Table 1, Figure 1D). The hinge region of the antibody Fab fragment is known to be flexible, and the crystal structure likely represents only one of multiple possible conformations. Thus, interface‐focused metrics such as the ICS, rather than RMSD, provide a more meaningful evaluation of predicted antibody Fab‐antigen complex models.

Despite these successes, no group was able to accurately predict the interface of target H1225 (Table 1). Even in top models, with the highest ICS scores of 0.625 or lower, the CCDs were not modeled correctly in terms of their Cα backbone conformations, nor in their binding interactions with the antibody. This is surprising given the successes in the predictions of our other two RSV G CCD–antibody targets. One possible reason could be the challenges in modeling a longer CDRH3 loop; however, this target had the shortest CDRH3 loop among the three (9 amino acids, compared to 10 and 12 in the others). Another possible reason could be a smaller interface area, but this target's interface area was similar to the others (Table 1). Although this antibody has a lower affinity for CCD than the other two, it is still in a nanomolar range, generally considered high. Thus, the reason for the poor predictive performance on this target remains unclear to us.

In summary, the CASP16 community has made exciting advancements in predicting the structures of antibody–antigen complexes. These advancements hold significant promise for the development of antibody‐based therapies to combat infectious diseases, cancer, inflammatory diseases, neurodegenerative diseases, and beyond.

### 2.2 Structure of the Bornavirus Polymerase Complex (CASP: H1220 and T1220S1, PDB: 9H1G, EMDB‐51765). Provided by Loic Carrique, Jonathan M. Grimes, and Jeremy R. Keown

Non‐segmented negative‐sense RNA viruses (nsNSV) from the family *Mononegavirales* are the causative agents of widespread human and animal disease, causing both localized epidemics and global pandemics. This large viral family contains over 900 species, including well‐known human pathogens like the Ebola virus, the Mumps virus, and the Rabies virus. Central to the lifecycle of nsNSV is the viral polymerase complex, which is formed from a hetero complex containing one copy of the Large protein (L‐protein) and two to four copies of the Phosphoprotein (P‐protein) [12]. During infection, the polymerase complex performs both replication and transcription of the viral genome, producing new copies of the viral genome and viral mRNA, respectively. For these processes, the L‐protein contains several domains: an RNA‐dependent RNA polymerase domain (RdRp) to synthesize RNA, a polyribonucleotidyltransferase domain (PRNTase) to cap mRNA, and a methyltransferase domain (MTase) to methylate the mRNA cap [12]. As part of this complex, the P‐protein performs a non‐catalytic chaperone role. The conserved and vital nature of these functional domains makes the L‐protein an ideal target for antiviral therapeutic development.

Bornaviruses are one family of nsNSV with an 8.9 kb genome, among the smallest in the family [13]. Mammalian Bornavirus 1 (BoDV‐1) is the prototype of the family causing infections and deaths localized to central Europe [14]. We used recombinant protein expression and purification to obtain a BoDV‐1 LP‐protein complex. Using single‐particle electron cryo‐microscopy, we reconstructed this complex at a resolution of 3 Å. We observed a single molecule of the 196 kDa L‐protein complexed with a tetramer of the 23 kDa P‐protein via an asymmetric interaction. While the RdRp and PRNTase formed a tightly associated core, the C‐terminal regions, including the connector domain, MTase domain, and C‐terminal domain, were at lower resolution due to their flexibility.

**FIGURE 2:**
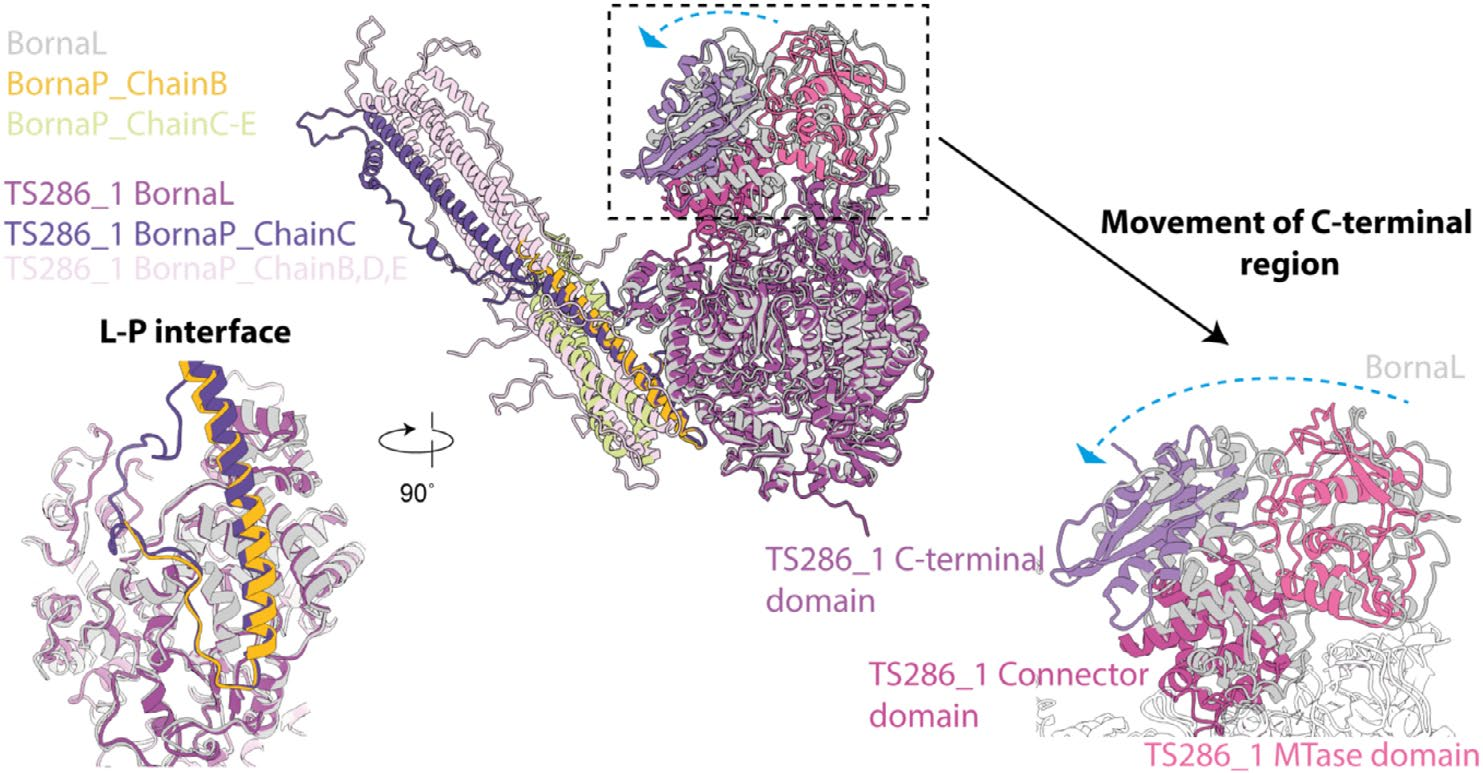
Comparison of the BornaLP complex with the prediction TS286_1. The experimentally determined structure of the Bornavirus Disease 1 LP (PDB: 9H1G, EMDB‐51765) complex is overlayed with the L‐protein (gray) and P‐protein (yellow, light green). Prediction TS286_1 was selected as a representative model for comparison. The L‐protein core (dark purple), the connector domain (light purple), the MTase domain (pink), the C‐terminal domain (lavender), and P‐protein (ChainB dark purple, ChainsC‐E pale pink) are shown.

The individual domain structure and approximate domain location of the individual polypeptides were correctly predicted for 68/71 L‐proteins and 69/71 P‐proteins. Many groups were able to correctly predict the experimentally confirmed helical‐kink region found in the BornaP tetramer [15, 16]. The interaction between the two proteins is mediated by one chain of the P‐protein tetramer. The interface is formed by one face of a helix and 12 residues from the P‐protein (ordered only in the L‐protein interacting chain) and the RdRp domain from the L‐protein. 19/71 predictions did not correctly identify the approximate location of the interface. Of the remaining 52, 34 of these were correctly able to recapitulate the molecular details of the interaction (Figure 2), while the others often positioned the helical interface where the C‐terminal peptide should be located. We have chosen H1220TS286_1 to exemplify this in the figure, though many predictions were equally good. Many predictions were unable to correctly orient the flexible C‐terminal region of the L‐protein, with most showing a (small) rotation of these domains towards the P‐protein (Figure 2). The predictions of this region varied widely, and given its biological role, it is likely it samples many of these orientations. Most models predicted that the N‐terminus of the L‐protein and the N‐ and C‐termini of the P‐protein would be disordered, in agreement with our experimental structure. In summary, 34 out of 71 models would allow us to draw similar biological conclusions to those determined experimentally in our model. Future developments in predicting the RNA path, binding of nucleotides, or conformational changes of the L‐protein have the potential to further increase the biological relevance of these predictions.

### 2.3 Bacteriophage T5 LTF pb1 (CASP: T1257, PDB: N/A). Provided by Alessio d'Acapito and Cécile Breyton

**FIGURE 3:**
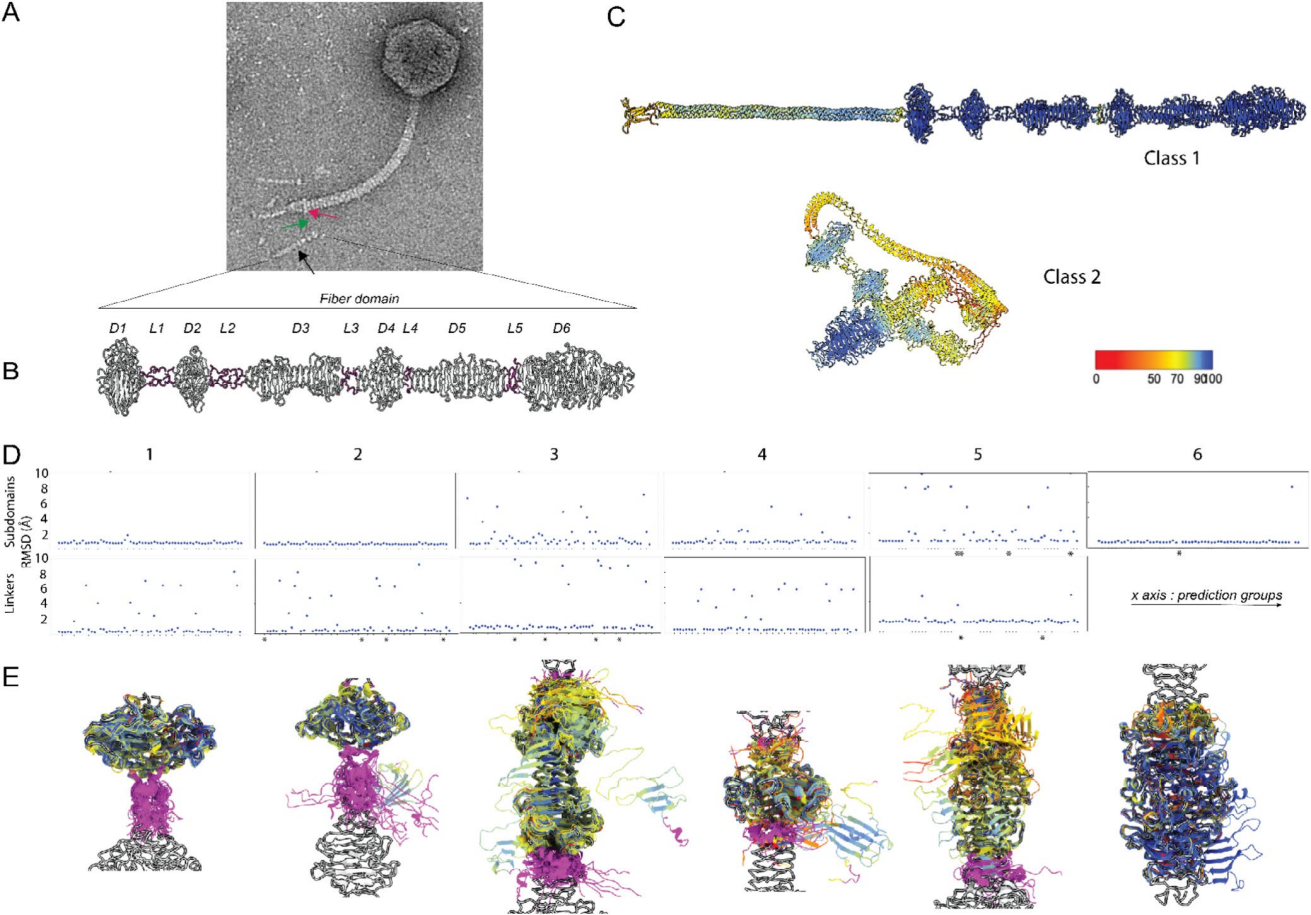
Predictions of T5 lateral L‐shaped fibers (LTF) pb1. (A) Negative staining EM image of a T5 virion. Arrows indicate the locations of the different domains of an LTF; red: Anchoring domain, green: Coiled‐coil domain; black: Fiber domain. (B) Experimental model of T5 pb1 fiber domain trimer (218–1263) obtained by single particle cryo‐EM analysis, with subdomains (D) colored in white and linkers (L) in magenta. (C) Examples of prediction from the two defined classes: Class 1: Good prediction of the overall shape and of the single domains; Class 2: Good single domain prediction, but globular overall shape of the protein. Predictions are colored by prediction confidence. (D) Quality of prediction of each subdomain of the pb1 fiber domain for the different groups. RMSD (in Å) values of structural alignment between the target and each of the 63 first predictions by domain (upper panel) and linkers (lower panel). Asterisks indicate values of RMSD out of range. (E) Superimposition by subdomain of the predictions, colored by prediction confidence (same color scale as in (C)), along with the neighboring linker (magenta). Target shown in white. The structural alignment is performed on the subdomain.

Bacteriophages (phages), viruses that infect bacteria, are the most abundant biological entities on Earth. All phages bear specific receptors that allow them to recognize their hosts and trigger infection [17]. Phage T5 is an *Escherichia coli*‐infecting phage characterized by a siphophage morphology: its DNA is enclosed in an icosahedral capsid, and host recognition receptors are located at the tip of its long, flexible tail (Figure 3A). The tail tip consists of a straight fiber [18] that carries the receptor‐binding protein pb5, which specifically recognizes the ferrichrome transporter FhuA on the outer membrane of *E. coli* [19]. To facilitate host recognition, each T5 particle also possesses three lateral L‐shaped tail fibers (LTFs) that mediate reversible binding to the sugar moiety of the host lipopolysaccharides [20], allowing the phage to walk on the surface of the bacteria until pb5 meets FhuA. Each LTF is made of a trimer of the protein pb1 that arranges into three main domains: an anchoring domain that clamps under the phage collar (1–40), a coiled‐coil domain that extends out from the phage (41–218), and a fiber domain (219–1263) (Figure 3A). The fiber domain is a remarkably rigid rod spanning 500 Å, composed of six subdomains (D1–D6) connected by rigid linkers (L1–L5) (Figure 3B). The last subdomain bears affinity to sugars (965–1263) and its structure has been solved previously, demonstrating that in the C‐terminus, the protein bears an auto‐cleaved chaperone domain (PDB ID: 5AQ5) [20].

**FIGURE 4:**
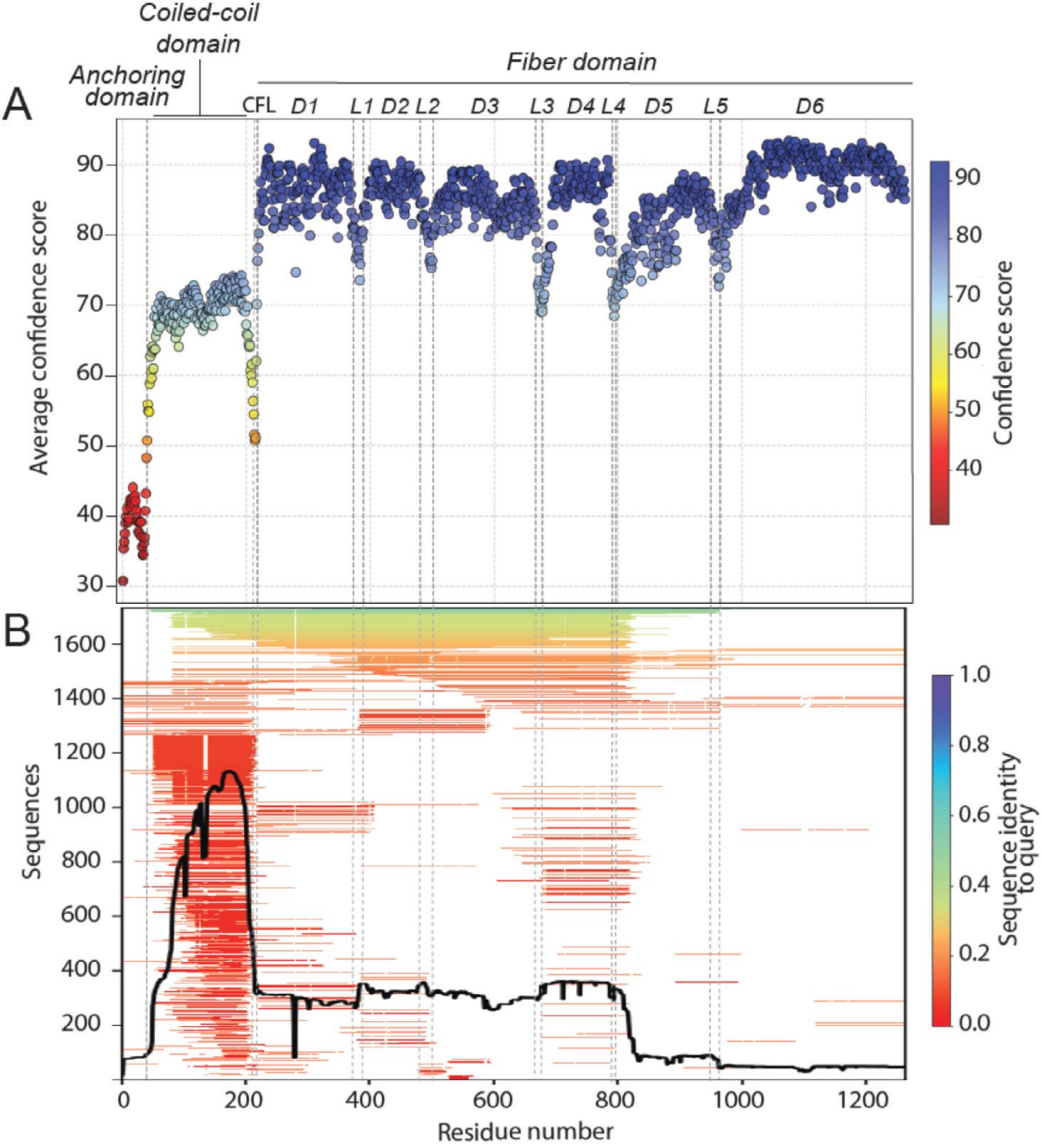
Prediction confidence. (A) Average per‐residue confidence level across all predictions, calculated from the “B‐factor” column of provided PDB files of the predicted models. Domains of full LTF pb1 are marked as in Figure 3A, with the addition of the coiled‐coil domain to the fiber domain linker (CFL). (B) MSA coverage of the pb1 sequence submitted to CASP16 obtained as an output of AlphaFold2. Dotted lines represent domains and linker limits both on (B) and (C).

We determined the structure of the anchoring and fiber domains of pb1 by analysis of single‐particle cryo‐electron microscopy (cryo‐EM) images of phage tails and submitted it to CASP16. The sequence provided to CASP16 included the structural domains, omitting the chaperone one. We categorized predictions into two groups: models that successfully predicted the straight conformation of the fiber domain (Class 1, 30 groups) and models that failed to do so (Class 2, 33 groups), displaying a fiber folded on itself, resulting in a globular overall shape (Figure 3C). This classification was based on a sharp increase in the provided Cα RMSD values between the target and the model (1.12–15.9 Å for Class 1 predictions, 76.7–135.7 Å for Class 2 predictions). We then performed structural alignment and RMSD calculations across all residues of each domain (Figure 3D,E). Next, we assessed the confidence level of the predictions by computing the average prediction confidence score across all residues, using the confidence score values stored in the “B‐factor” columns of the provided PDB files of the models (Figure 4A). Finally, we illustrated the MSA depth by obtaining a multiple sequence alignment (MSA) as an output of AlphaFold2 [21] to support our interpretations (Figure 4B).

None of the groups accurately predicted the anchoring domain, which had an average prediction confidence score below 50 (Figure 4A). This can be explained in part by the fact that *in phago*, the anchoring domain interacts with the phage collar and tube protein, resulting in a broken C3 symmetry that differentiates it from the rest of the protein. The MSA also revealed poor sequence coverage for this region (Figure 4B). The coiled‐coil domain was predicted as a coiled‐coil trimer by all groups, except for T1257TS293, T1257TS465, T1257TS079, and T1257TS196, with an average confidence score below 75 (Figure 4A). This sequence has the highest MSA coverage, with mostly low sequence identity (Figure 4B). While we did not solve the experimental structure of this domain, raw images and 2D classification of it on cryo‐EM images show that it consistently adopts a straight conformation, which was successfully predicted by most groups. In native LTFs, there is a variable kink between the coiled‐coil and fiber domains (Figure 3A), but none of the groups predicted this kink. Consistently, the sequence of this linker (~213–218) has the lowest prediction confidence of the whole domain (Figure 4A).

The fiber domain was the best‐predicted region, with an average confidence score of about 85, despite having shallow MSA coverage (Figure 4A,B). Within the fiber domain, the subdomains at the extremities (D1, D2, and D6) were the most accurately predicted (Figure 3D and Figure 4A,B), while central domains (D3, D4, and D5) showed more structural heterogeneity, as indicated by a more scattered RMSD plot (Figure 3D). Among these, D5 displayed the highest variability in predictions (Figure 3D,E) and the lowest confidence score (Figure 4A). This is likely because Class 2 predictions exhibited a globular shape, causing hinge points to concentrate at the center of the fiber domain, both on linkers and on subdomains that appear bent. Notably, the experimental structure revealed an uninterrupted β‐helix spanning from D5 to D6, which was accurately predicted by all Class 1 models. In contrast, most Class 2 predictions show a consistent breakage of the β‐helix at the level of L5 (Figure 3D,E), which is also predicted with lower confidence while being well structured in the experimental structure. D5 and D6 exhibited shallow MSAs, which may explain the lower prediction confidence for D5 (Figure 4A,B). However, D6 had higher confidence, likely because its structure was already available in the PDB [20].

Prediction confidence scores were systematically lower in the linkers (Figure 4A). They also exhibited greater structural heterogeneity than the subdomains, visible by more scattered RMSD plots (Figure 3D,E), also serving as the primary hinges for the “folding” observed in Class 2 models. In the experimental structure, L1, L2, and L4 are rigid random coils, while L3 and L5 are an α‐helix and part of the D5‐D6 β‐helix, respectively.

In conclusion, nearly half of the groups (30 out of 63) successfully predicted the straight conformation of the fiber domain, with most predictions correctly modeling the different subdomains. The main discrepancy, however, was in the overall folding of the fiber onto itself, with kinks in the linkers between subdomains. An additional parameter worth considering would be the calculation time, as this protein is rather large.

### 2.4 The DcmC Protein From the *dcm* Operon of *Methylobacterium extorquens* (CASP: T1246, PDB: N/A). Provided by Sabrina Bibi‐Triki, Françoise Bringel, and Benoît Masquida

Dichloromethane (DCM) is a major industrial solvent massively released in the environment and recognized as a potential carcinogenic pollutant. A few methylotrophs are able to degrade reduced one‐carbon compounds such as DCM as their sole energy and carbon source for growth [22]. Bacterial DCM‐degraders, including the reference *Methylobacterium extorquens* strain DM4, require the conserved DCM dehalogenase/glutathione S‐transferase encoded by the gene *dcmA*, which transforms DCM into two molecules of HCl and one molecule of formaldehyde [23]. Within the conserved *dcmRABC* gene cluster, only *dcmA* is essential for growth with DCM, unlike genes encoding for the repressor DcmR and two proteins of unknown functions, DcmB and DcmC.

In an effort to shed light on the *dcm* genes‐encoded proteins, we experimentally determined their start codon [24] before overexpression and attempted to crystallize the purified four proteins. Only DcmC yielded crystals suitable for diffraction studies. The 183 amino‐acid‐long DcmC protein presents no significant similarity to any protein with an identified function within the PDB and shows only limited homology to bacterial hypothetical proteins, providing little insight into its function.

We solved the structure of the DcmC protein at a resolution of 1.80 Å by Sulfur SAD (single‐wavelength anomalous diffraction) at the PX III beamline of the PSI‐SLS (Paul Scherrer Institute, the Swiss Light Source). The experimental electron density map showed an additional region with the size of a tripeptide, corresponding to a substrate spontaneously captured from the medium. This finding indicates that DcmC binds to a ligand, yet the ligand remains to be identified with confidence. The protein folds as a 10‐stranded antiparallel β‐barrel connected by loops of length between 3 and 20 amino acids. The access from one side of this robust β‐barrel is hindered by an α‐helix with a flexible hinge, while the opposite side of the β‐barrel seems to be accessible to the solvent. Four W and four F residues point towards the lumen of the barrel. Among those, two F residues belong to the closing N‐terminal α‐helix, contributing to the closure of the barrel. In the middle of this strong hydrophobic lumen remains an R residue (R89) that binds a chlorine ion. The overexpression of an R89A mutant yielded no protein, presumably due to the prominent role of this residue in the overall folding of the DcmC protein.

Structure‐based homology search by DALI [25] harvested different protein families sharing a 10‐stranded antiparallel β‐barrel domain with a maximum 13% sequence identity, indicating why the fold could not be identified by BLAST. Most of them are carriers of small organic ligands, like fatty acids (Fatty Acid Binding Protein, FABP), cholic acid, heme, or retinol [26, 27, 28, 29, 30, 31]. It is worth noting that in DcmC, the α‐helices that close one side of the barrel correspond to the N‐terminal end, whereas in most proteins identified by DALI, the barrel is closed by helices formed by a ~30‐residue L1 loop. DcmC, therefore, represents a topological variant of the FABP family.

The gap between the BLAST and DALI results made DcmC a compelling target for the CASP16 I structure prediction, as the relationship between its sequence and structure was unresolved in the databases. Nevertheless, the results from the CASP groups showed that this target was actually relatively easy to predict. The best models from the top 36 groups achieved a sequence‐independent local group alignment (LGA) score above 0.98, with only five groups scoring below 0.90 among the 63 that attempted to predict DcmC. A similar trend was observed for the local distance difference test (LDDT) scores, although among the top 12 models, the highest LGA did not always correspond to the highest LDDT.

**FIGURE 5:**
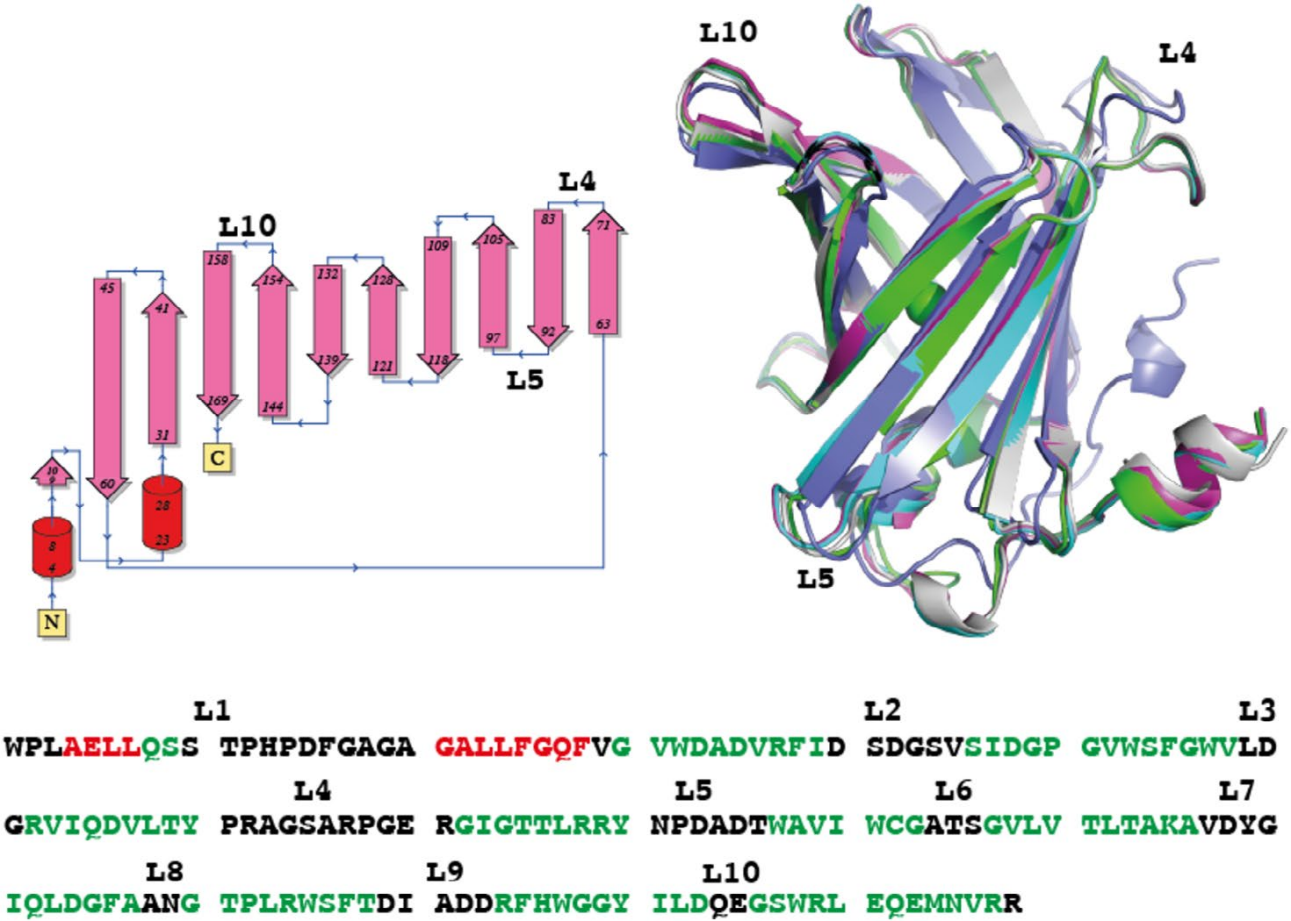
Superimposition of the top five structure models onto the DcmC target T1246 (green), T1246TS022_1 (Yang, cyan), T1246TS301_1 (GHZ‐MAN, gray), T1246TS052_1 (Yang‐server, purple), T1246TS284_1 (Unicorn, light pink), T1246TS075_1 (GHZ‐ISM, yellow), and the least accurate model, T1246TS167_1 (OpenComplex, slate). The topology, generated using PDBsum EMBL‐EBI, along with the sequence and the secondary structure (helix: Red; sheet: Green; loop: Dark) are also indicated for clarity.

In terms of local accuracy, the hydrophobic lumen of the barrel was very well predicted, with R89 correctly positioned at the center of the four tryptophan and four phenylalanine residues, despite the absence of the chlorine ion in the predicted model (Figure 5). The global distance test (GDT) highlighted the regions with lower accuracy. The N‐terminal subdomain, constituted by two successive α‐helices separated by a hinge that caps one side of the barrel, appears to be flexible, resulting in positional variability across most models. In addition, the N‐terminal end interacts with the L5 loop, which consequently adopts local conformations that deviate slightly from the conformation observed in the crystal structure. This behavior suggests a local concerted motion, which was not foreseen from the experimental structure alone. Moreover, in the L5 loop, the predicted models consistently extended the β‐sheets within the loop, leading to an offset of the Cα persistent across all models. As the GDT of the models decreased, we noticed that the flexibility of loop L4 increased. However, L4 also forms crystal packing contacts with L10 of a symmetry‐related monomer, and crystal packing contacts were also observed for N‐terminal α‐helices. Comparing the models with the target suggests that these regions may exhibit greater flexibility in solution, which does not necessarily reflect a decrease in the prediction quality.

### 2.5 F_420_ ‐Dependent Glucose‐6‐Phosphate Dehydrogenase (CASP: T1278, PDB: N/A). Provided by Gottfried J. Palm, Gerald Lackner, and Michael Lammers

The deazaflavin coenzyme F_420_ is utilized by bacteria and archaea in various redox reactions. Archaea use the cofactor during methanogenesis for hydride transfer from hydrogen to CO_2_‐derived single‐carbon intermediates. In *Mycobacterium tuberculosis* , the causative agent of tuberculosis, the cofactor plays a role in reduction reactions related to respiration, cell wall synthesis, as well as the activation of nitroimidazole prodrugs like delamanid. The redox potential of F_420_ /F_420_H_2_ (−340 mV) is lower than that of the more common redox cofactors NAD(P)H (−320 mV) and FADH_2_ (−220 mV). This enables reactions that are not easily accessible to many enzymes. In mycobacteria, the reduction of the cofactor is performed by an F_420_‐dependent glucose‐6‐phosphate dehydrogenase (Fgd). Similarly, Fgd from the thermophilic bacterium *Thermomicrobium roseum* catalyzes the oxidation of glucose‐6‐phosphate (in its ring form) to the lactone while reducing F_420_ to F_420_H_2_ [32]. Generally, Fgd belongs to the luciferase‐like domain superfamily that encompasses, besides FMN‐dependent luciferases responsible for bioluminescence, also flavin‐dependent monooxygenases involved in secondary metabolite biosynthesis or the above‐mentioned F_420_‐dependent hydride transferases. The broad substrate spectrum provides a useful basis to engineer enzymes suitable for new biotechnological purposes.

There are more than 100 sequence homologues of *T. roseum* Fgd deposited in the UniProt database with 50%–70% sequence identity and experimental structures in the PDB with up to 37% sequence identity. The closest homologues are dimers. While F_420_‐dependent homologues with their coenzyme bound were already known, substrate or product complexes had not been described. We have solved several structures at 2.2–2.5 Å resolution with and without substrate, but none with coenzyme. They include 54 crystallographically independent monomers, which exclusively form homo‐hexameric quaternary assemblies. The RMSD on Cɑ between the monomers is only 0.69 Å on average, independent of whether they are in the same or a different hexamer, and if they have ligands bound or not.

**FIGURE 6:**
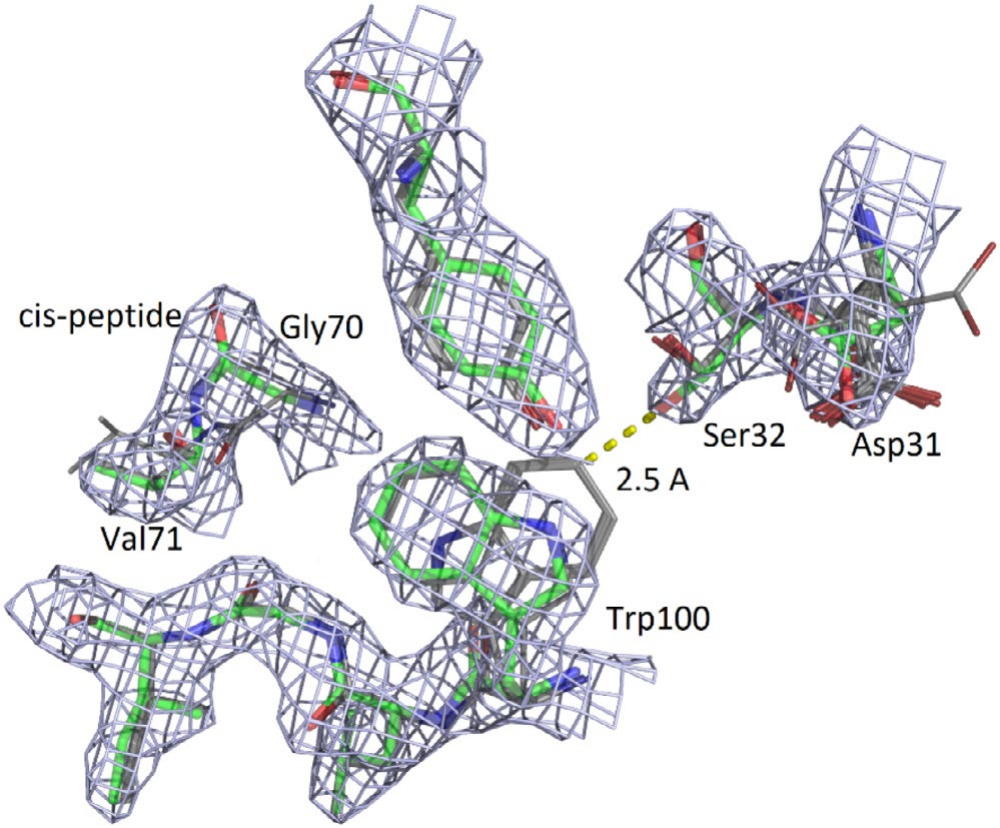
Superposition of an experimental model (shown in thick sticks in green, chain A of 9HAY) onto the 10 best predicted models (shown in thin sticks in gray, dark red, and dark blue). The rare non‐proline cis‐peptide bond, Gly70‐Val71, is correctly predicted by 9 out of 10 groups. Trp100 shows the most prominent deviations of the side chains' conformations (1 out of 10 groups predicted correctly). The 2mF_o_‐DF_c_ electron density map is shown in blue contoured at 1.0 RMSD.

In CASP16, only the monomers were to be predicted. As expected for a target classified as “easy” the main chain was predicted very well, with the RMSD on Cα being below 1 Å for 47 out of 62 groups. The best 10 predictions were inspected more closely, revealing that only 4 residues had Cα deviations greater than 1 Å. An interesting feature of the structure is a non‐proline cis‐peptide bond (Gly70‐Val71), which occurs rarely (< 0.1%) [33]. Although not unprecendented, it is encouraging that this bond was predicted correctly in 9 out of 10 cases (Figure 6). Cis‐peptide bonds are overrepresented in sugar‐binding proteins, to which Fgd belongs. Nevertheless, sugar binding is likely not the reason for this cis‐peptide bond, because it is conserved in related F_420_ enzymes, even if they do not have carbohydrate substrates. Regarding side chains, when several alternative conformations were observed in the X‐ray structures, all were considered valid. Only 10% of the side chains differed in at least one of the *χ* angles from all observed conformations. Most of these residues (70%) were on the surface or in the substrate binding site. Residues that were incorrectly predicted (or at least different from any observed conformations) included Arg (50%), Lys (25%), Pro (exo vs. endo), Glu, and Gln (approximately 20%). Trp100 was the only residue with an aromatic side chain in an incorrect conformation. A possible explanation for this could be the alternative side chain conformations observed for Ser32. In its predominant experimental conformation, Ser32 prevents the incorrect Trp100 conformation due to the hypothetical close contact of 2.5 Å (Figure 6). Although the occupancies were not refined, setting both conformers to 0.5 resulted in a B‐factor for the Trp100‐facing OG that was, on average, 4 Å^2^ lower. However, in the predicted models, Ser32 always adopted the alternate conformation with less density in the experimental structure, potentially leading to the incorrectly predicted Trp100 conformation.

In the predominant experimental conformation, Ser32 blocks the incorrect orientation of Trp100, likely due to a steric clash at a hypothetical distance of 2.5 Å (Figure 6). Although occupancies were not refined, setting both conformers to 0.5 resulted in a B‐factor for the Trp100‐facing OG that was, on average, 4 Å² lower. By contrast, in the predicted models, Ser32 consistently adopted the alternate conformation, which appears with lower density and occupancy in the experimental structure. This may have allowed the incorrect Trp100 conformation to be predicted.

### 2.6 Structure of a Cyclic Nucleotide‐Binding Protein From Gram‐Negative *B. bacteriovorus* (CASP: T1298, PDB: 9SFA). Provided by Matthew Jenkins, Henry Box, and Andrew L. Lovering

The predatory gram‐negative *B. bacteriovorus* has a lifecycle dependent upon invasion of gram‐negative prey [34]. Sequential stages of free‐swimming, prey recognition, surface adhesion, and commitment to invasion precede establishment of *Bdellovibrio* in the host cell periplasm, wherein it hydrolyses and assimilates host macromolecules. The elongated predator then septates into varying numbers of progeny that lyse the prey cell and search for a new host [35]. This bacteriocidal mechanism is independent of antimicrobial resistance status, prompting interest in its application against pathogens in agriculture, veterinary medicine, and human health [36].

This highly specialized niche requires *B. bacteriovorus* to tightly control the transitions between lifecycle stages [35, 36, 37]. Several second messenger‐based regulatory networks are known to contribute to this regulation. These include the highly developed *bis*‐3′,5′‐cyclic di‐guanosine monophosphate (CDG) network, with deletion of diguanylate cyclase enzymes shown to impair specific stages of the predation cycle [38]. Our interest was in the ubiquitous bacterial second messenger 3′,5′‐cyclic adenosine monophosphate (cAMP).

Beyond its classical role in catabolite repression, cAMP‐based regulation has been linked to processes associated with antibiotic resistance, including oxidative stress response and DNA repair [39]. Expression of catabolic enzymes is upregulated through the binding of cAMP to the catabolite gene activator protein, CAP, also termed cAMP receptor protein (CRP) [40]. CAP is a transcriptional activator with an N‐terminal nucleotide‐binding domain (NBD) and a C‐terminal DNA‐binding domain (CTD). In its apo‐state, CAP forms asymmetric dimers around a coiled‐coil interface between the α‐helix that links the NBD and CTD and the central C‐helix (PDB: 4N9H and 4N9I). The primary cAMP binding site is a moderately hydrophobic pocket that contains conserved arginine–serine (RS) and threonine–serine (TS) motifs. Cyclic nucleotide binding induces extension of a flexible hinge region within the C‐helix, causing rotation of the CTDs relative to the NBD dimer. This results in positional rearrangement of the CTD into a conformation that favors DNA binding [41].

**FIGURE 7:**
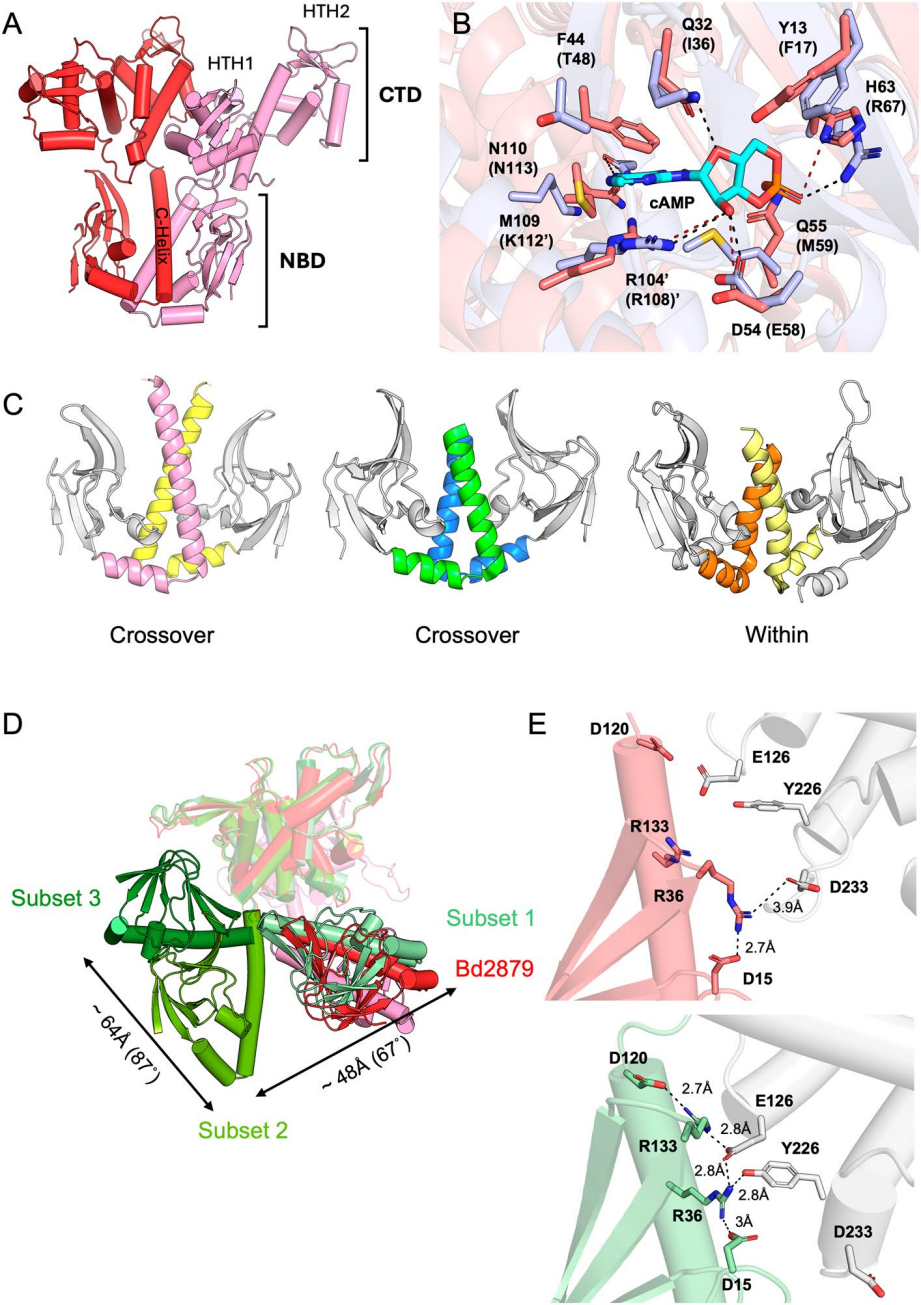
Crystal structure of Bd2879. (A) The Bd2879 asymmetric dimer, the N‐terminal nucleotide binding domain (NBD), and the helix‐turn‐helix (HTH1 and HTH2) of the C‐terminal domain (CTD) are labeled. (B) Alignment of the Bd2879 NBD (red) site with the cAMP‐bound site of the homologous Bd1971 NBD (blue). Residue labels are for Bd2879, with those of Bd1971 in brackets, denoting a residue on the opposing subunit. (C) The C‐helices (colored) of dimeric Bd2879 show the same crossover conformation as Bd1971, distinct from the standard CAP family conformation. *Left—*Bd2879; *Middle—*Bd1971 (PDB: 6HQ5); *Right*— *E. coli* CAP (PDB: 4N9H). (D) Predicted structures can be classified into three groups depending on the position of the NBD compared to the dimeric Bd2879 crystal structure NBD (red/pink) when models are superimposed using the CTDs (white). Group 1, represented by T1298TS274, aligns closely (pale green, RMSD =3 Å). Group 2, exemplified by T1298TS465 (medium green, RMSD = ~12.5 Å) and Group 3, represented by T1298TS475 (dark green, RMSD = ~16.6 Å), have NBDs that are rotated by ~67° and ~154° relative to the crystal structure, respectively. (E) Top—Close‐up view of the interactions at the top of the C‐helix in the Bd2879 crystal structure. Bottom—Interactions of the same interface in the overall top‐ranked model (T1298TS274).

The only published structure of a *B. bacteriovorus* cAMP‐binding protein is that of Bd1971, a CDG‐degrading enzyme of the EAL family that interacts with diguanylate cyclases (PDB: 6HQ5). Its N‐terminal NBD forms an asymmetric dimer around a central C‐helix, similar to CAP, but differs in interdomain architecture and interactions between subunits, as shown in Figure 7C [42].

Bd2879 is a 42 kDa putative transcription factor containing an N‐terminal NBD (residues 1–118), two putative DNA‐binding domains (residues 125–228 and 237–335, respectively), and a C‐terminal region with predicted disorder containing a zinc‐finger motif. To better understand how Bd2879 integrates a CAP‐like DNA‐binding function with a Bd1971‐like crossover NBD sensory domain, we solved its crystal structure at 2.88 Å resolution. The structure contains two copies, intriguingly arranged in an asymmetric dimer (Figure 7A). Residues S2‐D342 and S2‐E341 were resolved in chains A and B, respectively, while the C‐terminal residues 343–380 were presumed to be disordered. The Bd2879 NBD adopts a β‐roll and three α‐helix architecture, closely resembling the Bd1971 NBD. The NBD dimerization interface is similar to Bd1971, with a crossover‐style C‐helix (Figure 7C). The DNA‐binding domains, HTH1 and HTH2, adopt winged helix‐turn‐helix architectures. The Bd2879 NBD possesses a 16‐residue P‐loop (L52‐A67), with T65/S66 taking the place of the Bd1971 R67/S68 phosphate cradle and D54/Q55 replacing the ribose sensing E58/M59. Other residues are positionally conserved in Bd2879 vs. Bd1971 (Figure 7B), including the base‐capping residue R104 (R108), the nucleobase stabilizing N110 (N113), and 43‐LFQL‐46 of β4, equivalent to Bd1971 β5 (47‐LTIL‐50).

We grouped the predicted models for the 66 entries into several subsets based on their global Cɑ RMSD values when superposed onto our crystal structure. These RMSD values ranged from 2.2 to 2.8 Å (subset one, 18 models), between 8.1 Å and 15.3 Å (subset two, 34 models), and 17.7–19.4 Å (subset three, 7 models). The variation in RMSD values for the superposition of individual NBD and CTD domains was lower, ranging between ~1.5–3.7 Å and 1.2–3.6 Å, respectively. This suggests that the higher global RMSD values were primarily due to uncertainties in the predicted domain juxtaposition, likely caused by the flexibility of the loop linking the C‐helix to HTH1. When the full‐length models were superposed using the HTH domains as a fixed reference point, the three subsets could be seen to adopt distinct NBD positions (Figure 7D). The first subset with the lowest RMSD values, positioned the NBD in an orientation similar to that observed in the Bd2879 crystal structure (represented by T1298TS274). In contrast, for the second and third subsets (represented by T1298TS465 and T1298TS475), the position of the NBD is rotated by ~67° and ~154°, respectively.

The interdomain interface in the Bd2879 structure is formed at the loop that links the C‐helix to HTH1 (Figure 7E, top). Residue R36 of the β3/β4 turn interacts with D15 (β1–β2 loop) and D233 (HTH1/HTH2 linker loop), with a cluster of hydrophobic interactions between β3/β4 (NBD) and M117 (C‐helix), which lies ~4.2 Å from Y226. The top model is T1298TS274 (group one, global all‐atom RMSD 2.5 Å, NBD RMSD 1.59 Å, HTH1/HTH2 RMSD 1.43 Å, rank 1/66). The relative positions of the C‐helix, β3/β4 turn, and HTH1/HTH2 linker are similar to the Bd2879 structure (Figure 7E, bottom), although the observed interaction between R36 and D233 is not predicted. Instead, R36 is predicted to interact with D120 (C‐helix) and D125 (HTH1 α1), with R113 interacting with D15 and Y226. The relative distance between M117 at the top of the C‐helix and Y226 of the HTH1/HTH2 loop remains somewhat consistent at ~4.3 Å. In summary, this target highlights the ability of structure prediction methods to accurately reproduce the asymmetrical features observed in the experimentally determined structure.

### 2.7 Filaments of HD6 (CASP: T1219, PDB: 9R7N). Provided by Roman Kamyshinsky and Deborah Fass

HD6 is a member of a family of small, disulfide‐bonded proteins that defend the host against bacteria [43]. Unlike other defensins, however, HD6 does not appear to be directly microbicidal. Instead, the protein forms filaments that assemble into “nanonets” thought to entrap bacteria and prevent them from invading epithelial tissues [44]. Crystal structures of HD6 have been determined and revealed a two‐fold symmetric (PDB: 1ZMM), or slightly asymmetric (PDB: 1ZMQ), dimer of a twisted three‐stranded β‐sheet fold [45].

In one of the crystal structures (PDB: 1ZMQ), a filamentous arrangement of dimers can be traced within the crystal, serving as the basis for models for the HD6 nanonet filaments [44, 46]. Along this embedded filament, adjacent dimers form an intermolecular β‐sheet‐like interaction with an amino‐terminal β‐strand from each dimer. Another way of describing this interaction is as follows. Due to the twist of the two interacting three‐stranded β‐sheets in each dimer, the six strands nearly form a barrel. However, instead of the barrel closing on itself, the amino terminus of the next near‐barrel contributes a β‐strand. This donated strand is not long enough to close the first barrel, but as the strand also participates in the β‐sheet of the second dimer, it bridges the β‐sheets of the first and second dimers, resulting in a continuous β‐sheet that spirals through the dimers along the filament. The dimers themselves also spiral around a central axis.

**FIGURE 8:**
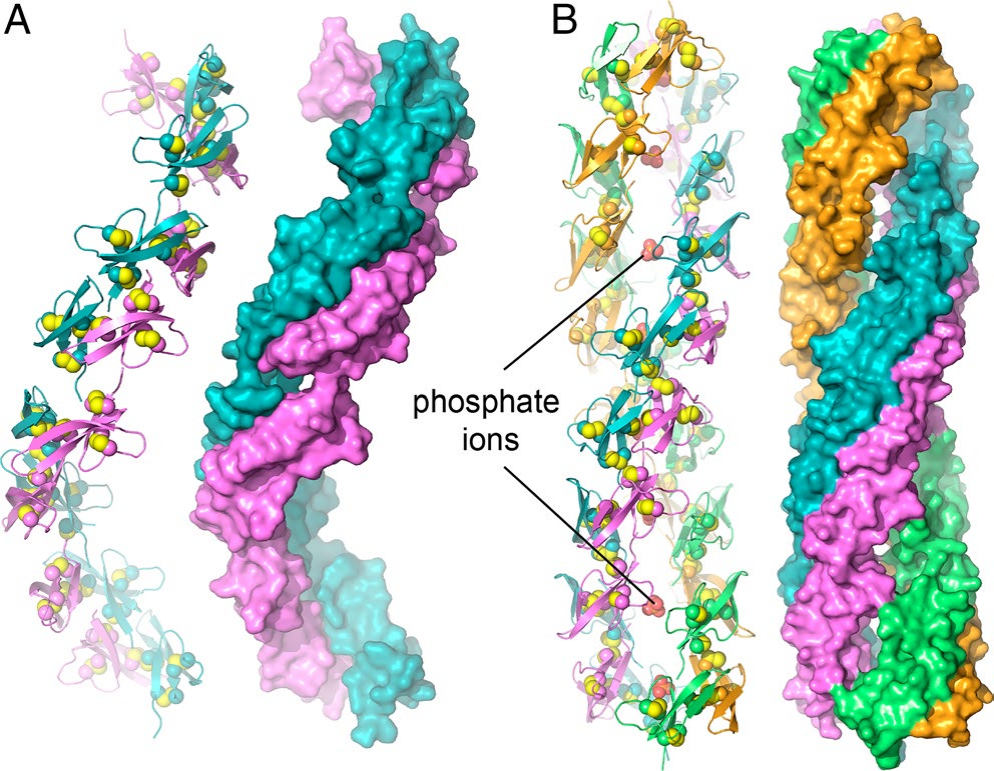
Comparison of a CASP helical model for HD6 with the experimental structure of the HD6 inner filament. (A) Cartoon and surface representations of the T1219v1TS221_1o prediction. Cysteine side chains, participating in disulfide bonds, are shown as spheres with yellow sulfurs in the cartoon. One subunit of each dimer is colored violet, and the second subunit is colored teal. (B) The cryo‐EM structure of HD6 filaments shows two dimers approximately facing one another (171°) across the filament axis. A phosphate is positioned near the axis at each level of the filament. The four protomers in the two dimers at each level are colored violet, teal, light orange, and lime.

The CASP predictions for spiral HD6 structures resembled the filament model derived from the crystal structure (Figure 8A), with some, such as T1219v1TS221o.pdb, maintaining a 90° rotation between successive sets of four HD6 protomers, that is, two dimers along the filament, as required by the screw axis of the crystal symmetry. Other predictions yielded a similar spiral but with slight deviations from the crystal angle, such as the 88° rotation between sets of four HD6 protomers in T1219v1TS4251o.pdb. The individual dimers observed in the crystal structure and CASP predictions closely resembled dimers in the cryo‐EM filaments, with backbone RMSD values of about 0.9 Å, making them suitable for fitting into the cryo‐EM map. However, neither the crystal structures nor the predictions provided a hint of for the unusual higher‐order assembly of dimers in the cryo‐EM structure. The true self‐assembly modes of HD6 filaments observed experimentally and described below were not captured in the CASP models.

Reproducing a previous protocol [46], HD6 filaments were generated in vitro. These filaments were analyzed using cryo‐EM, yielding a structure at 3.6 Å resolution. One important insight from the HD6 cryo‐EM structure was that the core of the HD6 filaments contained two dimers, not a single dimer, at each level around the filament axis (Figure 8B). This arrangement was not present in the previous HD6 crystal structures or the CASP predictions. The four HD6 protomers at each level of the filament in the cryo‐EM structure were arranged in two sets of dimers, approximately facing one another across the filament axis but related by a rotation of 171° rather than 180° around the axis. Moreover, one dimer was displaced by about 1.5 Å along the filament axis relative to the other. Were it not for these two significant breaks in symmetry, the HD6 filaments would have displayed D2 symmetry. As the CASP predictions did not anticipate the second set of dimers in the filament, the symmetry breaks were not a feature of the predictions.

A physiologically important discovery from the HD6 cryo‐EM structure was that the filament contains phosphate ions along its axis. Each set of four HD6 protomers was seen to accommodate phosphate in a basic cavity formed by the four copies of His5, Arg7, and the NH^3+^ group at the polypeptide terminus, which all pointed inward. This finding implies that the presence and distribution of phosphate or related ions in the luminal environment of the intestine may determine the site and rate of HD6 nanonet formation. Lack of knowledge of phosphate coordination may have further complicated the CASP prediction of the HD6 filament composition and structure.

Previous studies of HD6 by negative stain transmission EM revealed filaments microns in length [46] that appeared wider than the original, spiraling dimer model based on the crystal structure. Moreover, the remarkably apparent persistence length of the filaments suggested a particularly robust mode of supramolecular assembly. In addition to the core of the filament described above, the cryo‐EM structure revealed two sets of additional HD6 dimers spiraling around the central, phosphate‐coordinating dimers (not shown). This feature was not suggested by the crystal structure or previously predicted, but the outer spirals likely stiffen and support the filaments. As the outer spirals associate heterogeneously with the inner filament of the HD6 cryo‐EM structure, their presence and helical parameters would likely be very difficult to predict.

In summary, none of the CASP predictions captured the duplication of the dimer‐based spiral seen in the cryo‐EM structure. The predictions that successfully suggested filaments as the HD6 assembly mode were limited to the dimer spirals resembling those derived from the previous HD6 crystal structures.

### 2.8 Human Twisted Gastrulation 1 (CASP: T1201 and T1201o, PDB: 8BWD), and Its Complex With Growth Differentiation Factor 5 (CASP: H1202, PDB: 8BWL). Provided by Tomas Malinauskas and Christian Siebold

Bone Morphogenetic Proteins (BMPs) are secreted extracellular proteins that orchestrate the embryonic development and homeostasis of all multicellular organisms. Dimeric BMPs initiate signaling by binding to two types of transmembrane receptors: BMP type 1 (BMPR1) and type 2 (BMPR2) serine/threonine kinases. Bridging both receptors triggers the phosphorylation of BMPR1 by the constitutively active BMPR2, which, in turn, activates transcription factors. Interactions between BMPs and their receptors are controlled by extracellular modulators [47, 48, 49]. Disrupting BMP‐modulator interactions leads to developmental disorders.

Twisted Gastrulation 1 (TWSG1) is a secreted glycoprotein that, together with Chordin family members, is required for BMP gradient formation during dorsal‐ventral patterning of embryos [49]. Human TWSG1 is composed of 223 amino acid residues, including 24 cysteines. Our search for structural homologues using HHpred [50] revealed no homologues of TWSG1 in the PDB. At the start of our studies on TWSG1, in the pre‐AlphaFold era (around 2017), this protein remained an enigma. Its domain architecture, folds, and interactions with binding partners (BMPs and Chordin, a modulator of BMP signaling) were either completely unknown or poorly characterized. We determined the crystal structure of full‐length human TWSG1 at 2.63 Å resolution using de novo heavy‐atom phasing, as well as the structure of the N‐terminal domain of TWSG1 in complex with a BMP, Growth Differentiation Factor 5 (GDF5), at 1.96 Å resolution [49].

**FIGURE 9:**
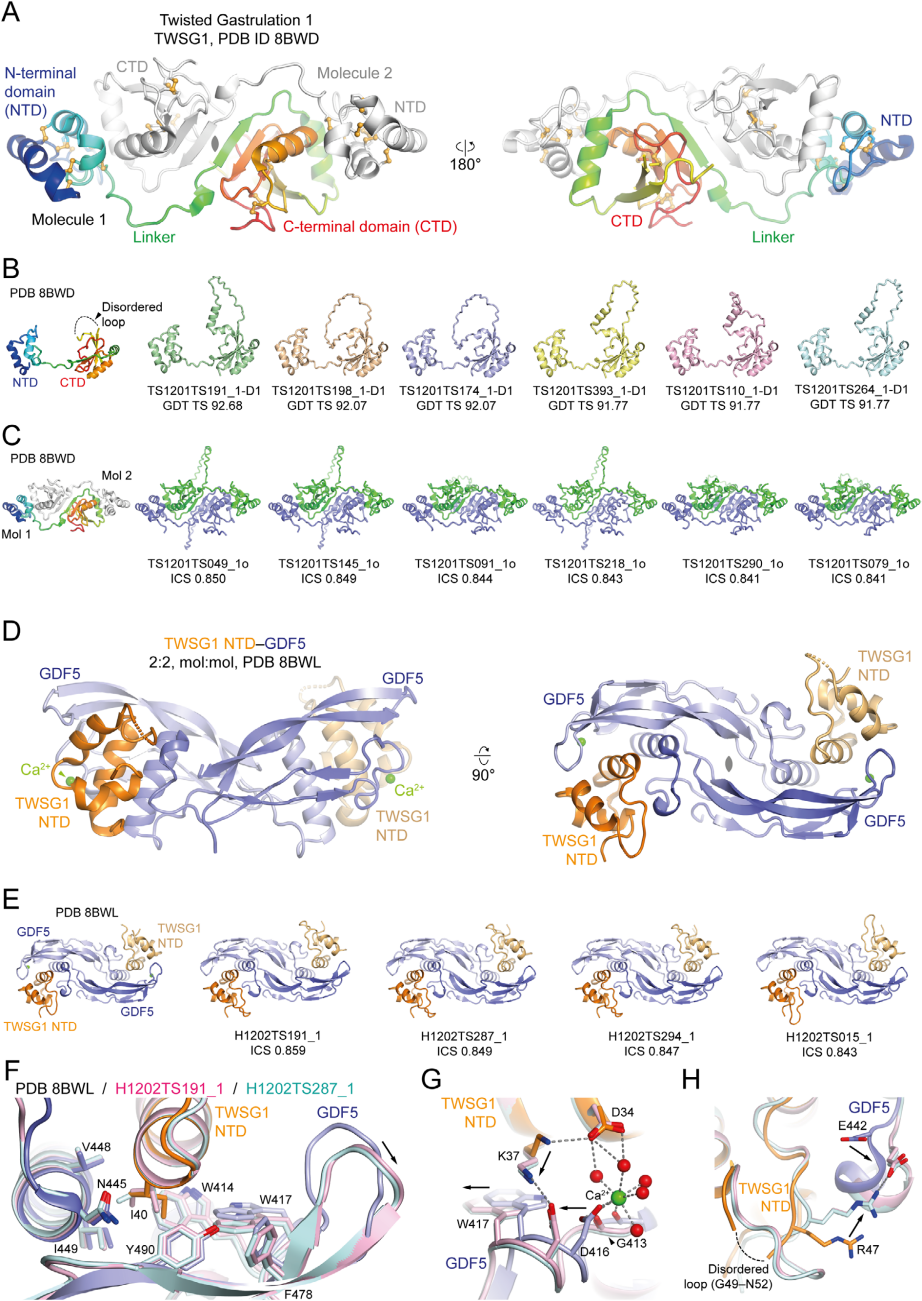
Comparison of the TWSG1 and TWSG1 NTD–GDF5 crystal structures to the highest‐ranking CASP16 models. (A) The crystal structure of the TWSG1 dimer, with one protomer depicted in rainbow coloring (N‐terminus in blue; C‐terminus in red) and the second protomer colored gray (PDB: 8BWD). Disulfide bonds are shown as orange balls‐and‐sticks. (B) Comparison of the TWSG1 monomer (PDB: 8BWD) to the six highest‐ranked prediction models (CASP T1201; GDT_TS 91.77–92.68). The disordered loop, which was not visible in the crystal structure but is present in the predictions, is indicated. (C) Comparison of the TWSG1 dimer (PDB: 8BWD) and the six highest‐ranked prediction models (CASP T1201o; ICS 0.841–0.850). (D, E) The crystal structure of dimeric GDF5 in complex with two TWSG1 NTDs (PDB: 8BWL) (D) and a comparison to the highest‐ranking prediction models (CASP H1202; ICS 0.843–0.859) (E). (F–H) A comparison of the GDF5–TWSG1 NTD crystal structure with the two highest‐ranking models reveals discrepancies at the amino acid side‐chain level, such as the rotamer of TWSG1 Ile40 (F), or the predicted salt bridges between TWSG1 Lys37 and GDF5 Asp416 (G), and GDF5 Glu442 and TWSG1 Arg47 (H), which are not present in the crystal structure. In (F–H), the crystal structure is colored as in (D), while the prediction models are shown in pale cyan and pink.

TWSG1 crystallized as a dimer in two different crystal forms, which is consistent with its dimerization in solution, albeit relatively weak [49]. The structure of TWSG1 revealed a modular architecture comprising an α‐helical N‐terminal domain (NTD, Cys26‐Arg80) connected by an extended linker to a mixed α/β C‐terminal domain (CTD, Pro87‐Phe223) (Figure 9A). The NTD of TWSG1 folds into a compact bundle of three α‐helices, stabilized by seven disulfide bonds. The CTD of TWSG1 is stabilized by five disulfide bonds and contains a β‐sheet composed of five antiparallel β‐strands forming its core.

We compared the full‐length structure of TWSG1 (PDB: 8BWD) to the CASP16 predictions. All 90 predicted models of the TWSG1 monomer correctly suggested that TWSG1 is composed of two cysteine‐rich domains separated by a linker (Figure 9B). Of these 90 models, 70 correctly predicted all 12 disulfide bonds in TWSG1. The top predictions also accurately captured the relative orientation of the NTD and CTD. We remain curious whether these top groups first modeled the TWSG1 dimer and then presented a single protomer for CASP16.

Interestingly, 5 out of 90 models largely predicted the folds and secondary structures of the NTD and CTD correctly, but all 5 models completely lacked disulfide bonds. This highlights the limitations of some models at the level of amino acid side chains and their rotamers. The absence of disulfide bonds in these TWSG1 models points to a potential opportunity for improvement: considering the extracellular location of the target could help with side‐chain accuracy, as extracellular human proteins, such as TWSG1, are often stabilized by disulfide bonds.

67 out of 74 models of the TWSG1 dimer are largely correct, as indicated by the juxtaposition of two antiparallel β‐strands from two TWSG1 CTDs and the recapitulation of the NTD‐CTD interface observed in crystal structures (Figure 9A,C). We were pleasantly surprised by the prediction of the TWSG1 dimer, given that TWSG1 tends to dimerize only partially and only at relatively high protein concentrations in solution (~0.1–1.0 mg/mL) [49].

In addition, to understand how TWSG1 interacts with BMP ligands, we determined the crystal structure of the TWSG1 NTD in complex with GDF5 (Figure 9D). The disulfide‐linked GDF5 dimer binds to two TWSG1 NTD molecules, which are related by a non‐crystallographic pseudo‐two‐fold symmetry axis [49]. Here, we compare the structure of the TWSG1 NTD in a complex with GDF5 (PDB: 8BWL) to CASP16 predictions (H1202).

The high‐resolution crystal structures of GDF5, both in isolation and in complexes with its binding partners, are available to train the latest protein modeling programs [48, 49, 51, 52, 53, 54]. As expected, the GDF5 dimer and its secondary structures were accurately predicted in 74 out of 77 prediction models. 70 out of 77 prediction models recapitulated the key interaction between Ile40 of the TWSG1 NTD and a hydrophobic pocket of GDF5 (Figure 9E). Curiously, none of these largely correct 70 models had the same rotamer of TWSG1 Ile40 as observed in the crystal structure (Figure 9F). Some residues lining the side of the hydrophobic pocket of GDF5 (such as Trp417) were displaced in the predicted models compared to the crystal structure by a couple of angstroms. One reason for this discrepancy might be the presence of calcium in the crystal structure. The calcium and its coordinating water molecules bridge Gly413 and Asp416 of GDF5 to Asp34 of the TWSG1 NTD in the crystal structure (Figure 9G). In contrast, even in the highest‐scoring prediction models, Asp416 of GDF5 forms a hydrogen bond with Lys37 of the TWSG1 NTD. We do not see this interaction in the crystal structure. Similarly, we do not observe predicted interactions between TWSG1 Arg47 and GDF5 Glu442 in the crystal structure (Figure 9H).

In summary, top‐scoring CASP16 prediction models globally recapitulated key hydrophobic interaction surfaces between the TWSG1 NTD and GDF5; however, the details of the interactions at the level of amino acid side chains and their rotamers differed from the crystal structure.

### 2.9 The Structure of the Hemoglobin‐NbE11 Nanobody Complex (CASP: H1204, PDB: 8VYL). Provided by Rhys Grinter and Daniel R. Fox

Hemoglobin is an abundant protein found within erythrocytes, which most commonly consists of a heterotetramer of α and β subunit dimers, with each subunit carrying the iron‐containing cofactor heme. Using these heme groups, hemoglobin binds oxygen (O_2_) and transports it throughout the body. Due to its abundance, the heme groups of hemoglobin constitute the largest source of iron within the human body [55]. Many bacterial pathogens have evolved strategies to scavenge heme from host hemoglobin. In Gram‐negative pathogens, this often occurs via the direct extraction of heme from hemoglobin by TonB‐dependent transporters (TBDTs) in the bacterial outer membrane [56]. Reduced concentrations of hemoglobin in the blood or the presence of hemoglobin outside of the circulatory system can be indicative of disease states, such as beta thalassemia [57]. Hemoglobin‐binding proteins like nanobodies can be used to detect the presence and concentration of hemoglobin and are invaluable in the development of diagnostics for these conditions. In addition, hemoglobin‐binding nanobodies aid in the investigation of the molecular mechanisms of hemoglobin targeting TBDTs [56].

**FIGURE 10:**
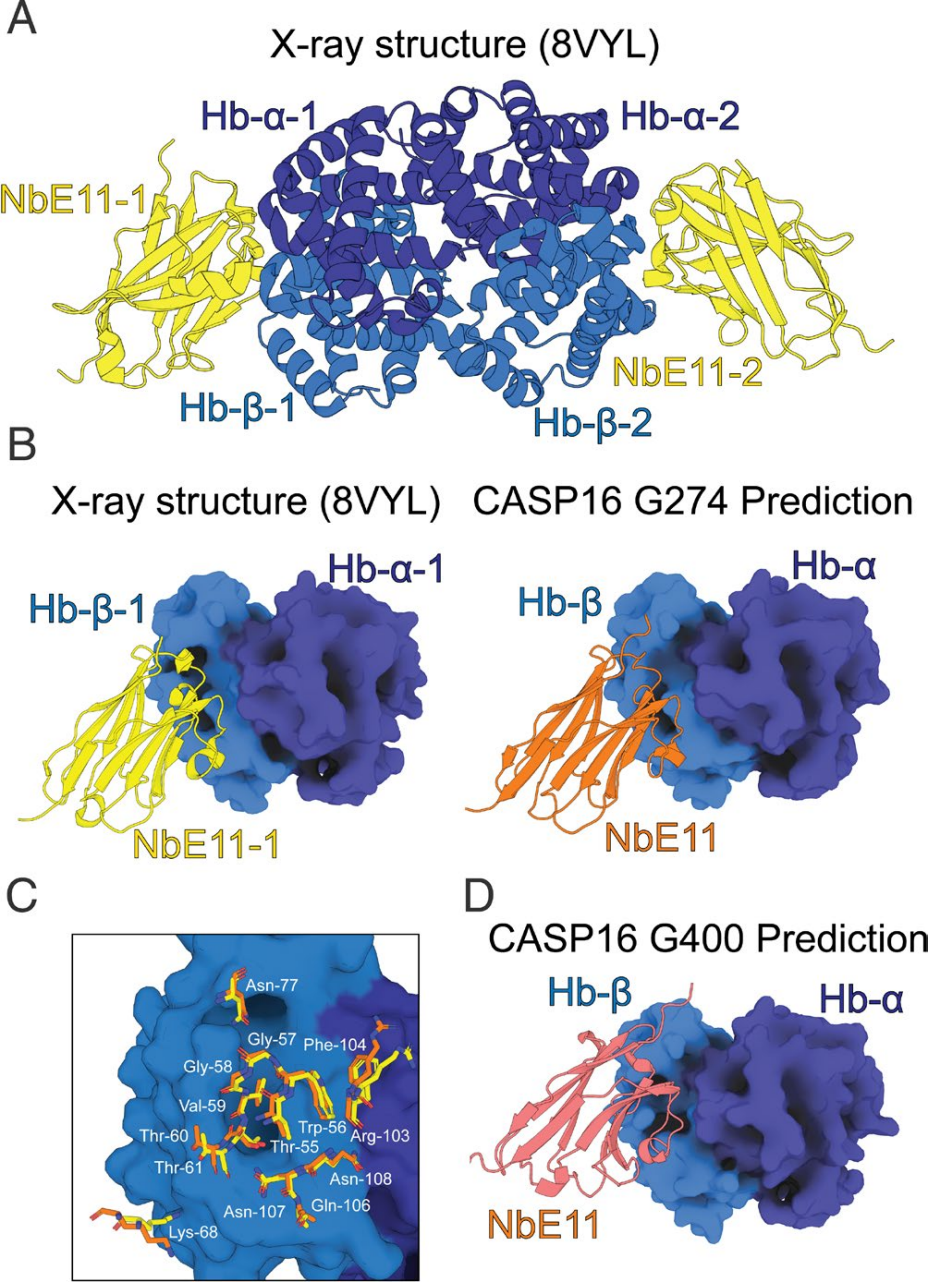
Comparison of NbE11‐hemoglobin crystal structure with top CASP16 predictions. (A) The crystal structure of the NbE11‐hemoglobin complex. (B) A comparison of the orientation of NbE11 bound to αβ‐hemoglobin in the crystal structure (left) and the model submitted by G274 (right). (C) A superimposition of NbE11 residues interacting with hemoglobin in the crystal structure (yellow) and the G274 model (orange). (D) The incorrect orientation of NbE11 in the model submitted by G400.

Previous work identified NbE11 as a high‐affinity hemoglobin‐binding nanobody that can be used to detect hemoglobin in human stool samples and demonstrated that it lacks cross‐reactivity to other vertebrate hemoglobin species [58]. Nanobodies are small, single‐domain antibody fragments derived from the heavy‐chain‐only antibodies found in camelids [59]. Their simplified structure allows for the isolation of stable, soluble antigen‐binding domains that function independently. To enhance the usefulness of NbE11, we solved the crystal structure of the nanobody bound to adult (αβ) hemoglobin and determined the affinity and thermodynamics of binding [57] (Figure 10A). This structure shows that NbE11 binds across the α and β subunits of hemoglobin, primarily binding the β‐subunit (Figure 10B). The asymmetric unit of the structure consisted of a hemoglobin tetramer bound to two copies of NbE11, with the two αβ‐hemoglobin dimer‐nanobody subcomplexes related by non‐crystallographic symmetry [57].

We submitted the NbE11‐hemoglobin structure to CASP16, as it remains challenging to model nanobodies and antibodies in a complex with their target antigens with high accuracy. We had previously attempted to model this complex using current state‐of‐the‐art protein structure prediction programs AlphaFold3 and Chai‐1, which reported good performance for antibody–antigen modeling [60, 61]. However, they failed to accurately predict the complex in our case. Nanobodies and antibodies possess three and six hypervariable loops, respectively, termed complementarity‐determining regions (CDRs), which facilitate antigen binding. CDRs can undergo rounds of affinity maturation via somatic hypermutation to create and improve antigen‐binding functionality [62]. Many structure prediction models are informed by MSA, which derive structural information from evolutionary relationships across protein families. Because of the variable nature of CDR loop generation, MSAs provide little assistance in the prediction of antibody/nanobody‐antigen structures [63], increasing the difficulty of predicting these complexes [64]. Moreover, CDR loops can also undergo conformational change upon epitope binding and are intrinsically flexible, further complicating modeling efforts [65]. Previous entries of nanobody/antibody–antigen complexes to CASP14 and CASP15 were challenging targets, with no groups able to perfectly model either of the complexes comparable to the experimental structures [6, 7]. However, prediction of nanobody/antibody–antigen complexes is improving [60, 61, 63, 65, 66].

When assessing the accuracy of the CASP16 models, we noted that while our NbE11‐hemoglobin tetramer structure was pseudosymmetrical due to the specific nature of the crystal packing, most H1204 entries exhibited C2 symmetry, which is also likely biologically relevant. As such, when comparing our structure to the CASP16 models, we limited our analysis to the alignment of one NbE11‐αβ dimer pair. While most groups were successful in predicting the structure of the hemoglobin αβ‐dimer and NbE11 components separately, only the top‐ranked model (based on model‐target RMSD) from the Kozakovvajda group (G274) modeled NbE11 bound to hemoglobin in the same position and orientation as our experimental structure (Figure 10B). The positioning of sidechains at the interface between NbE11 and hemoglobin was also highly accurate, confirming that the structure of the complex was successfully predicted (Figure 10C). This was reflected in the high ICS (0.820), QS (best) (0.902), TM (0.942), and DockQ (0.847) scores for this model, in addition to it recording the lowest RMSD of the complex (2.561 Å). The next six models (G400, G204, G196, G456, G52, G79; ranked by QS) all modeled NbE11 in a similar position relative to hemoglobin. The position of NbE11 relative to hemoglobin was broadly similar to the experimental structure. However, the orientation of NbE11 was incorrect, indicating failure to accurately model the complex (Figure 10D). Consistent with this, the scores were generally worse for these models (ICS [0.337–0.565], QS [best] [0.646–0.678], TM [0.443–0.861], DockQ [0.569–0.628], RMSD [3.870–5.609 Å]). The Kozakovvajda group model had a similar LDDT score (0.871) to the models of Groups 3–7 (0.815–0.838), which is consistent with most top groups accurately modeling the αβ‐dimer and NbE11 components separately, meaning that global LDDT was not a good indicator of the correct placement of NbE11 in the complex.

In summary, the Kozakovvajda (G274) was able to accurately predict both the correct binding interface and orientation of the NbE11b‐hemoglobin complex. This is a significant achievement, given the difficulties in modeling nanobody/antibody–antigen complexes. While accurate modeling of nanobody/antibody–antigen complexes is still challenging, the successful modeling of the NbE11‐αβ hemoglobin complex in this CASP round indicates that we are close to a general solution to this problem. The accurate modeling of antibody–antigen complexes is important for the computational design of de novo antibodies as it allows for rapid in silico screening to identify the best designs [67]. The advances demonstrated in CASP16 will help to realize the potential of this technology, which has significant medical and biotechnological applications.

### 2.10 The Nanobody Nb48 Bound to the Coiled‐Coil Domain of BILBO1 (CASP: H1244; PDB: N/A): Provided by Kim Abesamis and Gang Dong

*Trypanosoma brucei* is a parasitic protist causing sleeping sickness (human African trypanosomiasis) and nagana (a disease affecting livestock) in sub‐Saharan Africa. To survive and thrive within its host, the parasite has developed unique adaptations, including the flagellar pocket, a specialized membrane invagination located at the base of its single flagellum [68]. This structure serves as the exclusive site for endocytosis and exocytosis in *T. brucei* , playing a critical role in nutrient uptake, waste removal, and immune evasion [69]. The integrity and function of the flagellar pocket are maintained by a collar‐like cytoskeletal structure encircling its neck, known as the flagellar pocket collar (FPC) [70].

BILBO1 is a key scaffold protein within the FPC, essential for its assembly and structural integrity [71, 72]. The protein comprises four distinct domains: a globular N‐terminal domain, two central EF‐hand motifs, an extended coiled‐coil domain, and a C‐terminal leucine zipper [73]. Our previous studies have demonstrated that BILBO1 forms antiparallel dimers through its coiled‐coil domain, which subsequently assemble into long filaments via interactions between the leucine zipper motifs of neighboring dimers [74]. These filaments can further associate laterally, forming tightly packed bundles that contribute to the structural robustness of the FPC [75].

**FIGURE 11:**
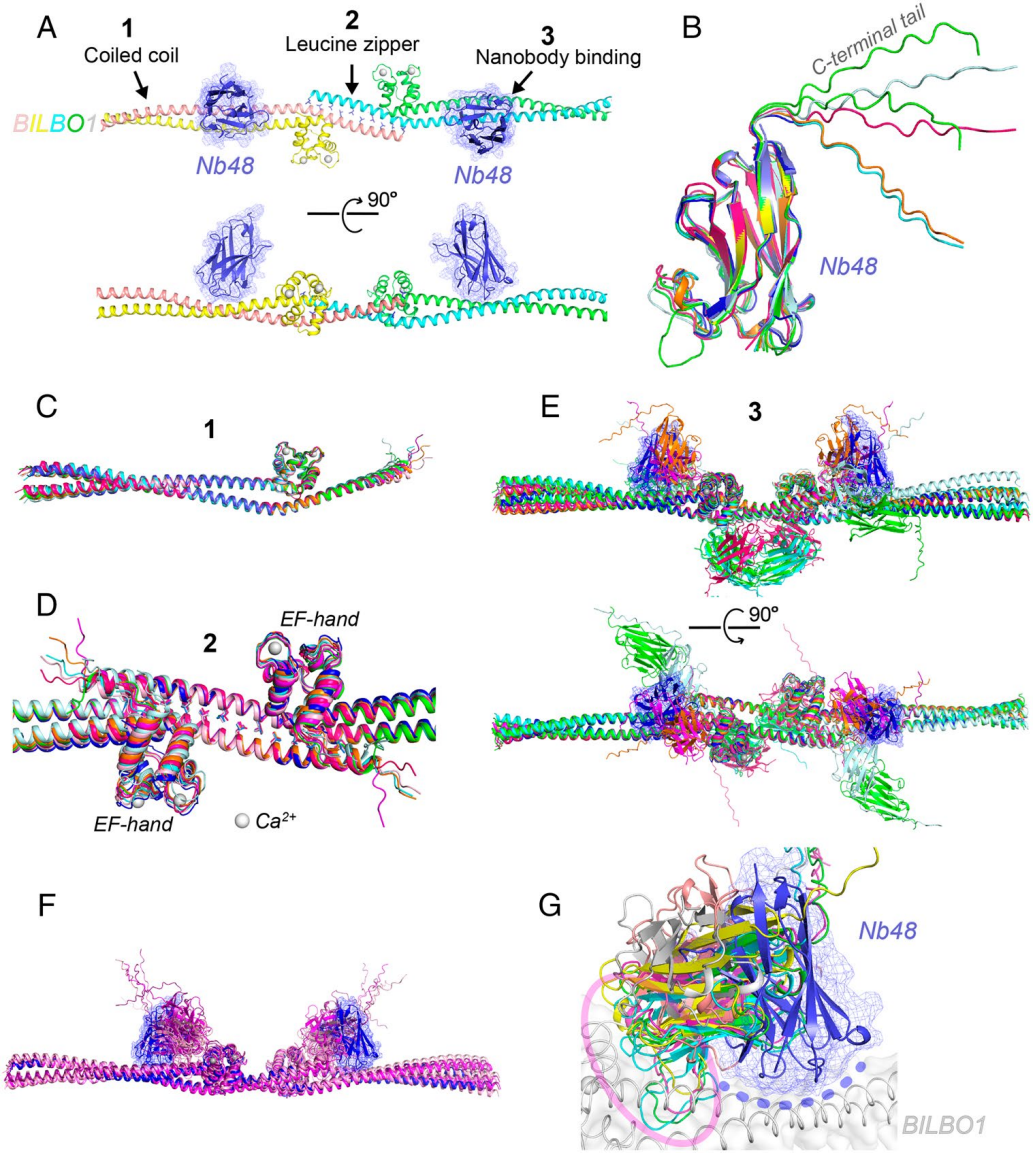
Structural analysis of the nanobody Nb48 in complex with BILBO1. (A) Crystal structure of the Nb48‐TbBILBO1 complex, showing the filament junction of BILBO1 (residues 179–334 and 461–587) bound to Nb48 (blue, with mesh representation). The structure reveals three intermolecular interactions: (1) the antiparallel coiled coil of BILBO1, (2) the antiparallel leucine zipper of BILBO1, and (3) the nanobody binding site on BILBO1. (B) Superposition of all Nb48 models in the top‐scoring predictions from each category in the “Per Target Analysis” table of the CASP16 results with the crystal structure of Nb48 (blue). The C‐terminal tail of the nanobody is shown to be flexible. (C, D) Superposition of all top‐ranked CASP16 predictions within the antiparallel coiled‐coil region (C) and the leucine zipper junction (D) of BILBO1. (E) Two orthogonal views of the CASP16 top‐scoring models superimposed on the BILBO1‐Nb48 crystal structure (blue). (F) Superposition of the six non‐redundant CASP16 predictions with an overall RMSD < 15 Å (purple) onto the BILBO1‐Nb48 crystal structure (blue). (G) Enlarged view of the Nb48 binding site from (F). The experimentally determined Nb48‐BILBO1 binding interface in the crystal structure is highlighted with a dashed blue arch. Notably, in all CASP16 predictions with an overall RMSD < 15 Å, Nb48 is predicted to interact with the globular EF‐hand motif (purple oval) rather than the actual binding site.

A nanobody named Nb48 was developed to specifically target BILBO1, inducing rapid cell death, likely by disrupting FPC assembly and function [76]. However, the precise molecular mechanism underlying this inhibition remains unclear. Recently, using a series of binding assays with Nb48 and various BILBO1 truncations, we mapped the Nb48 binding site to the coiled‐coil region adjacent to the leucine zipper junction of BILBO1. Furthermore, we determined a high‐resolution (2.2 Å) crystal structure of Nb48 in complex with the BILBO1 junction, which includes the EF‐hand motif, the leucine zipper, and part of the coiled‐coil domain (residues 179–335 and 461–587) (Figure 11A). The structure of the complex revealed two copies of Nb48 bound to a symmetrically arranged pair of BILBO1 coiled coils, featuring three key intermolecular interactions: the antiparallel coiled coil, the leucine zipper, and the Nb48‐BILBO1 binding interface.

We submitted the BILBO1 junction–Nb48 complex to CASP16 for protein multimer prediction and analyzed the results using the “Per Target Analysis” table available on the CASP website. For each prediction category, we selected the top‐ranked model and assessed the accuracy of the nanobody Nb48. The results demonstrated that the structure of Nb48 was predicted with high accuracy (Figure 11B). Additionally, all top‐scoring models successfully predicted the antiparallel coiled‐coil structure of BILBO1, with an overall Cα RMSD of 1.0–1.9 Å (Figure 11C). Similarly, these models correctly predicted the leucine zipper connecting two neighboring BILBO1 coiled‐coil dimers, with an RMSD of 1.1–2.5 Å (Figure 11D). However, the positioning of Nb48 varied significantly across predictions, with most placements differing drastically from the nanobody's actual binding site in the crystal structure (Figure 11E). The best RMSD score between any predicted model and the target structure was 11.9 Å (Bhattacharya group/369). We further compared the top six non‐redundant predictions with overall RMSD values below 15 Å (Figure 11F). While these predictions positioned Nb48 in a similar region on the BILBO1 junction complex, a closer examination revealed that none of them accurately identified the true epitope located on the coiled‐coil domain. Instead, the nanobodies were consistently packed against the globular EF‐hand motif (Figure 11G).

Our analysis of the CASP16 predictions highlights the challenges in accurately predicting recognition sites of antibodies and nanobodies on target antigens. We attribute this difficulty to two main factors in our case. First, the epitope is situated in a highly confined region on an extended coiled coil, making it inherently complex to recognize and model. Second, the interaction between the nanobody and the coiled coil involves a sophisticated network of electrostatic and hydrophobic interactions, engaging numerous residues from four loops of Nb48 and both helices of the BILBO1 coiled coil. This intricate interplay of interactions further complicates accurate prediction.

### 2.11 The Cryo‐EM Structure of the LRRK2:14‐3‐3_2_ Complex (CASP: H1258, PDB: 9CI3, EMD‐45609). Provided by Juliana Martinez Fiesco and Ping Zhang

**FIGURE 12:**
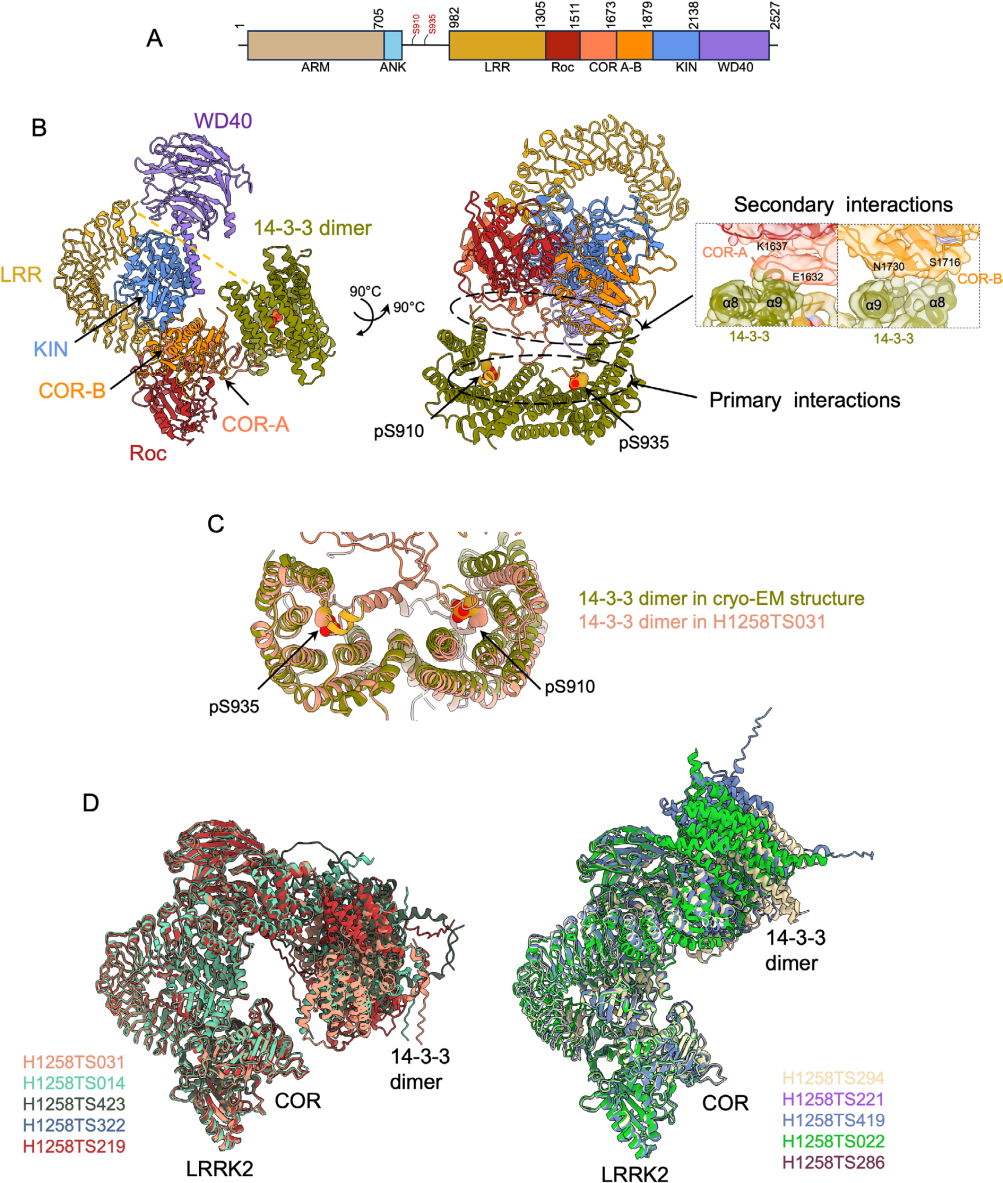
The complex between LRRK2 and 14‐3‐3 proteins. (A) Schematic representation of the LRRK2 domain organization. Residues S910 and S935 are highlighted in red, as they serve as 14‐3‐3 binding sites upon phosphorylation. (B) Cryo‐EM structure of LRRK2: 14‐3‐3_2_ complex (unresolved residues 1–906 dotted yellow), colored as in (A), shown in two different orientations. The primary and secondary contact regions between LRRK2 and 14‐3‐3 are indicated (right), respectively. In the primary interaction, the LRRK2 phosphorylation sites, pS910 and pS935, bind to 14‐3‐3 substrate binding grooves (right). In the secondary interaction, the LRRK2‐COR domain engages with α‐8 and α‐9 helices of the 14‐3‐3 dimer. (C) The top 10 predictions closely resemble the LRRK2: 14‐3‐3 primary interaction. For clarity, only the top prediction (H1258TS031) is shown, aligned with the cryo‐EM structure by the 14‐3‐3 dimer. (D) Top 10 predictions grouped by structural similarity in two different groups. Showing the relative position of the 14‐3‐3 dimer to the COR domain within the predicted LRRK2/14‐3‐3 complexes. None of these predictions recapitulate the LRRK2‐COR: 14‐3‐3 interface shown in the cryo‐EM structure (A).

Leucine‐rich repeat kinase 2 (LRRK2) is a large (2527 residue) multidomain protein involved in critical cellular processes including signal transduction, cellular trafficking, and autophagy [77, 78]. It is composed of several functional domains. The central feature of LRRK2 is its kinase domain, which enables the phosphorylation of various proteins and thus regulates numerous signaling pathways. LRRK2 also contains in tandem the Ras of complex protein (Roc) and the C‐terminal of Roc (COR) domains, conferring it with GTPase activity. Additionally, LRRK2 includes several domains that serve as regulatory or protein–protein binding domains; among them are a C‐terminal WD40 domain and an N‐terminal regulatory armadillo (ARM), ankyrin (ANK), and leucine‐rich (LRR) repeats (Figure 12A). The structural understanding of LRRK2 has significantly advanced in recent years, revealing important insights into its monomeric and dimeric forms, as well as its interactions with microtubules [79, 80, 81].

The biological significance of LRRK2 is underscored by its link to neurodegenerative diseases, most notably Parkinson's disease (PD). Mutations in the LRRK2 gene are a significant cause of PD [82, 83, 84, 85]. Most PD‐associated mutations lie within the catalytic domains, often leading to kinase hyperactivity. This hyperactivity disrupts cellular processes regulated by LRRK2, ultimately leading to impaired autophagy, mitochondrial dysfunction, and neuroinflammation [86, 87, 88], although the precise mechanism linking mutations in LRRK2 to PD remains to be fully elucidated. Consequently, LRRK2 is a promising target for therapeutic intervention in PD, with ongoing efforts to develop inhibitors of its kinase activity. Understanding the precise mechanisms of LRRK2 function and regulation could open novel avenues for therapeutic development in neurodegenerative disorders.

LRRK2 is regulated by 14‐3‐3 proteins, which influence LRRK2 stability, localization, and kinase activity through binding [89, 90]. Furthermore, several PD‐associated mutations have diminished 14‐3‐3 interaction, which correlates with increased kinase activity [91, 92, 93]. 14‐3‐3 proteins are ubiquitous, widely expressed dimeric scaffolds that bind primarily through conserved sequences containing phosphoserines or phosphothreonines in their target proteins [94, 95]. Occasionally, a secondary interaction can be established by interfaces between the globular domains of the target proteins and an additional surface in 14‐3‐3 [96]. In LRRK2, a cluster of potential 14‐3‐3 primary binding sites, including pS910 and pS935, is located before the LRR domain. Despite the well‐established interaction between LRRK2 and 14‐3‐3, the exact molecular details of their interaction remain unknown. As part of our efforts to investigate LRRK2 regulation by 14‐3‐3, we determined the structure of the LRRK2/14‐3‐3 complex by cryo‐EM.

Our study shows that the complex is formed between a LRRK2 monomer and a 14‐3‐3 dimer (Figure 12B). The N‐terminal region (residues 1–906), including the ARM and ANK domains, was absent from the density map, suggesting it is flexible. LRRK2 adopts a conformation similar to the previously reported kinase‐inactive LRRK2, with the LRR domain positioned to occlude substrate access to the kinase active site. 14‐3‐3 assumes the canonical cup‐like shape with two phospho‐client‐binding grooves that establish the primary interaction with LRRK2 pS910 and pS935 residues. Additionally, a unique, previously unidentified secondary binding interface is observed between the LRRK2 COR domain and the 14‐3‐3 dimer (COR‐A and COR‐B, Figure 12B). This secondary interface is functionally relevant as it helps to position the LRR domain, thereby reinforcing the inhibited state of LRRK2 and evidencing the mechanism by which 14‐3‐3 inhibits LRRK2 kinase activity.

For CASP16, participants were provided one copy of LRRK2 and two copies of 14‐3‐3 as input for multimer modeling. Out of 64 predictions generated, 62 reported binding between the LRRK2 and a 14‐3‐3 dimer. Of these, 54 predictions correctly identified the 14‐3‐3 phospho‐binding grooves within the 14‐3‐3 dimer, reflecting the conserved nature of this interface. However, most predictions failed to localize the correct LRRK2 phospho‐sites involved in the interaction (pS935 and pS910). Only the top 10 predictions successfully captured both correct binding grooves of 14‐3‐3 and the interacting LRRK2 segments, aligning with our cryo‐EM structure (Figure 12C). Despite this success, a major limitation of the predictions was the lack of phosphorylation modeling. Since 14‐3‐3 binding is phospho‐dependent, omitting these modifications likely impaired accurate interface modeling.

This underscores the challenge of accurately modeling posttranslational modifications in current prediction methods. Additionally, none of the predictions resolved the secondary binding interface between LRRK2 and 14‐3‐3 (Figure 12D). This may be due to the relative rarity of these secondary interactions, which likely occur within the context of the primary binding event. The inability to predict the secondary interface is reflected in the maximum prediction scores, which were generally low (ranging from 0.0 to 0.6) across all metrics, including Precision, Recall, IPS, QS‐Glob, and QS‐Best. These results highlight that predicting multimeric interactions, particularly those involving posttranslational modifications and non‐canonical secondary interactions, remains a significant challenge for current prediction models.

### 2.12 Rabbit Dystrophin–Glycoprotein Complex DGC (CASP: H0272, H1272, and H2272, PDB: 9C3C). Provided by Shiheng Liu, Tiantian Su, and Z. Hong Zhou

Duchenne muscular dystrophy (DMD) is one of the most common inherited muscle‐wasting diseases, impacting approximately 1 in every 3500–5000 male newborns globally [97], which ultimately leads to fatal cardiac and/or respiratory failure between 20 and 40 years of age, even with optimal care [98]. Despite being first described in the 1860s, little was known about the cause of DMD until the discovery of the dystrophin gene [97, 99]. Systematic biochemical characterizations of the dystrophin‐associated proteins have established their interactions with the sarcolemma, cytoskeleton, channels, and signaling proteins, either directly or indirectly, forming the dystrophin‐glycoprotein complex (DGC) [100, 101, 102, 103]. Ironically, despite the identification of most associated factors, mechanistic understanding of the DGC's function as a “molecular shock absorber” for the sarcolemma and its numerous disease‐associated mutations remains very limited. This could be largely due to a lack of atomic structures of the protein complexes involved.

**FIGURE 13:**
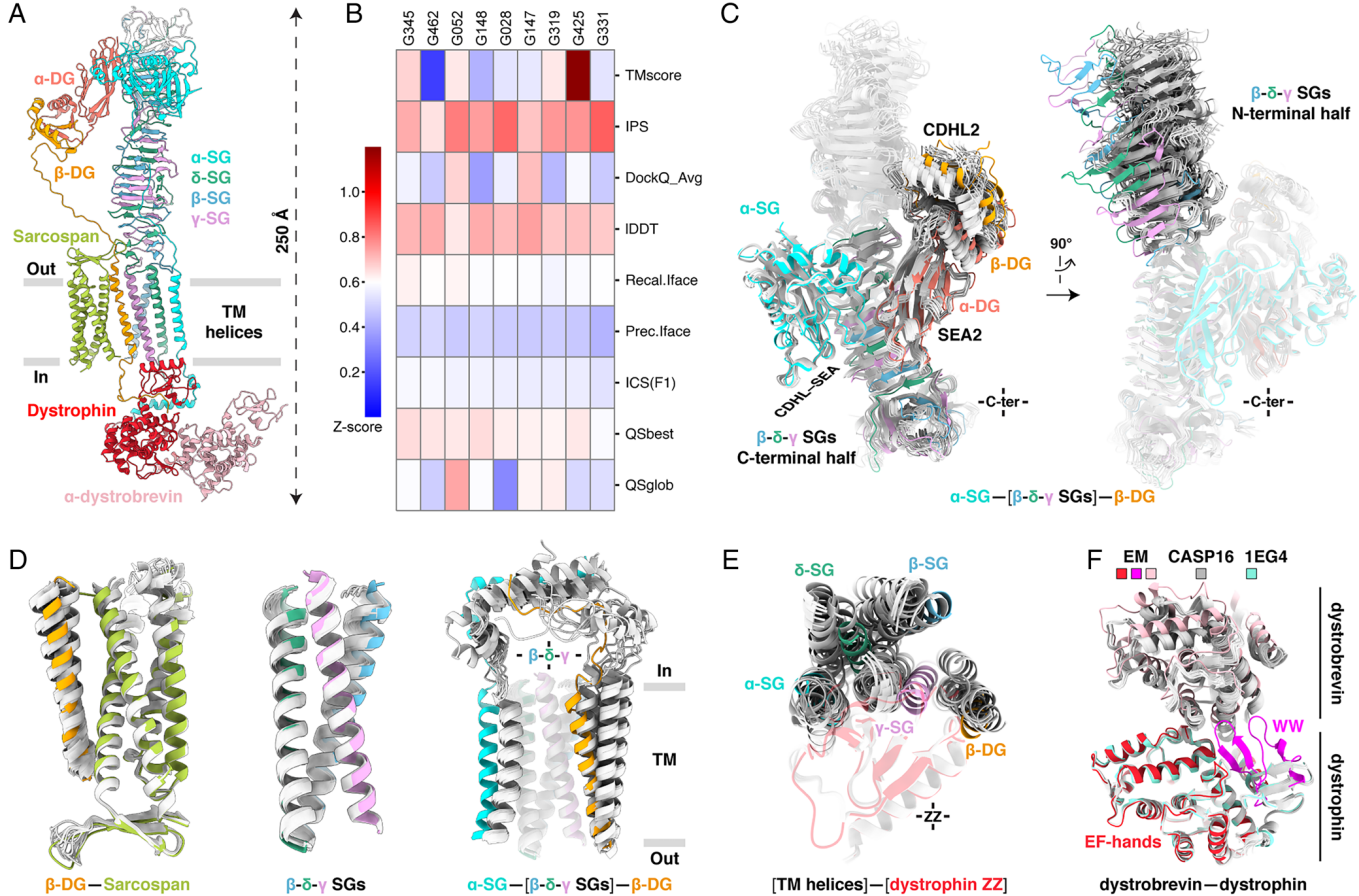
Comparison of CASP16 predictions of DGC with the experimental structure. (A) Atomic model of DGC derived from cryo‐EM (PDB: 9C3C), shown in cartoon representation. (B) Z‐score heatmap illustrating the relative performance of selected models across evaluation metrics. Models from 9 groups (deduplicated from 12) were identified through manual curation as capturing all five essential interfaces observed in the experimental DGC structure. Each column represents a model, and each row corresponds to a scoring metric. *Z*‐scores were calculated as *Z* = (*x* − *μ*)/*σ*, where *x* is the respective model's raw score, *μ* is the mean, and *σ* is the standard deviation of scores across all 36 deduplicated, first‐submitted models from Phase 1 predictions. This standardization enables assessment of each selected model's performance relative to the full distribution. Color gradients represent *Z*‐score values, transitioning from 0 (blue) to 1.2 (deep red), with warmer colors indicating progressively stronger performance across metrics. (C–F) Structural comparison of key interfaces between the cryo‐EM structure (colored as in (A)) and the predicted models (in light gray): (C) β–γ–δ SG β‐helix interacting with α‐SG and α/β‐DG; (D) Transmembrane helices; (E) Transmembrane helices' variability relative to dystrophin's ZZ domain; and (F) dystrophin–α‐dystrobrevin heterodimer. All nine selected models were included in Panels (C, D, and F). For improved clarity, two models from G052 and G148 were omitted in Panel (E). In Panel (F), the dystrophin WW domain is highlighted in magenta, and PDB 1EG4 in light green.

Using cryo‐EM, we determined the atomic structure of native DGC directly enriched from the skeletal muscle membrane [104], containing nine core components: dystrophin, dystroglycan (DG) subcomplex (α‐DG and β‐DG), sarcoglycan (SG) subcomplex (α‐SG, β‐SG, γ‐SG, and δ‐SG), sarcospan, and α‐dystrobrevin. The DGC comprises five essential protein–protein interaction clusters that define its overall architecture (Figure 13A). First is the extracellular β–γ–δ SG trimer that forms a curved β‐helix. Second, this β‐helix interacts with the [cadherin‐like (CDHL)–SEA] modules of α/β‐DG and α‐SG. Third are the transmembrane helices that are arranged with β‐DG positioned between sarcospan and the β–γ–δ SGs, while α‐SG associates with β–γ–δ SGs on the opposite side. Fourth is the juxtamembrane fragment of β‐DG and SGs that interacts with the cystine‐rich ZZ domain of dystrophin. And finally, the fifth is the cytoplasmic dystrophin–α‐dystrobrevin heterodimer formed by their cystine‐rich regions.

Despite the success of recent machine learning‐based structure prediction methods, as evidenced in CASP15, the DGC's large size and compositional/conformational complexity are still expected to pose a considerable challenge for the multimeric structure prediction tools. Our manual assessment of Phase 1 predictions on the first‐submitted models revealed 12 groups from five labs: MULTICOM (G331, G425, G319, G345), NKRNA‐s (G028), Yang (G052/G456), Guijunlab (G148/G264/G312), Zheng (G147, G462), which successfully recapitulated all five essential DGC interfaces. Among the top five models ranked by ICS score, only MULTICOM_human (G345, #2) and Zheng (G462, #3) captured all five interfaces. To pinpoint the most reliable indicators of model quality, a *Z*‐score heatmap was computed across all metrics and models (Figure 13B), revealing IPS as the top‐performing metric (predominantly reddish, *Z*‐scores 0.65–0.84), followed by IDDT and QSbest.

Regarding the extracellular part, all 12 groups accurately predicted the N‐terminal half of the curved β‐helix formed by the β–γ–δ SG trimer and its interface with the two CDHL–SEA modules from α‐SG and α/β‐DG (Figure 13C, left). In contrast, the predicted C‐terminal half displayed a pronounced tilt relative to the cryo‐EM structure (Figure 13C, right), reflecting challenges in modeling the correct curvature shaped by a conserved four‐residue insertion in β‐SG [104]. Within the membrane region, the arrangement of transmembrane helices, β–γ–δ SG bundle (Figure 13D, left) and β‐DG–sarcospan complex (Figure 13D, middle) was generally well predicted. However, the transmembrane conformations of β‐DG and α‐SG deviated from the cryo‐EM structure (Figure 13D, right), likely stemming from subtle differences in cytoplasmic juxtamembrane interactions.

For the cytoplasmic part, the juxtamembrane region of β‐DG was generally docked to the cysteine‐rich ZZ domain of dystrophin, but the positioning of the ZZ domain relative to the transmembrane helices varied across models (Figure 13E). This variability is likely due to the fact that the ZZ domain is connected to the membrane via a few long, flexible linkers from both β‐DG and SGs and that no comparable interface has been structurally characterized to date. These findings imply inherent structural dynamics between cytoplasmic dystrophin and the membrane, potentially contributing to the complex's mechanical flexibility and resilience. Although the EF‐hand‐mediated interaction between dystrophin and α‐dystrobrevin was frequently captured, none of the models accurately predicted the interaction between dystrophin's WW domain and α‐dystrobrevin's EF1 (Figure 13F). In all predictions, the WW domain adopted the conformation observed in the crystal structure of dystrophin's cysteine‐rich region (PDB: 1EG4), underscoring the heavy reliance of current structure prediction algorithms on existing PDB templates.

## 3 Conclusions

This article presents a comprehensive analysis of selected CASP16 targets, focusing on both structural accuracy and biological relevance. The authors of the experimental structures highlighted key features that were successfully reproduced in models, while also discussing the limitations of current prediction methods. Overall, the ability to predict three‐dimensional protein structures continues to be impressive, with many challenging targets modeled to a high degree of accuracy.

Notably, we observed successes in predicting structures of large protein complexes, although these complexes were generally smaller than those in the previous CASP round. In several cases of protein complex predictions, including targets H1220, T1246, H0272, H1222, H1223, H1272, and H2272, the predictions were sufficiently accurate to enable meaningful biological interpretation.

Some predicted structures also likely reflected functionally meaningful conformational states. For example, the antibody–antigen complexes, which are important for therapeutic development, offer particularly valuable insights into the progress made in structure prediction. As shown in H1204, H1222, and H1223 cases, accurate modeling of nanobody and antibody binding interfaces is becoming more feasible, though challenges remain for certain epitopes and interaction modes, such as H1225 and H1244.

Alongside these successes, CASP16 also revealed areas in need of improvement. A persistent challenge is the accurate placement of side chains, especially in functional or interface regions, as seen in targets T1201, T1278, and H1202. However, some predictions, notably H1204, showed surprising accuracy at nanobody interfaces not observed in previous rounds.

Posttranslational modifications, such as phosphorylation, remain difficult to model reliably, as illustrated by target H1258. Accurate prediction of these modifications is crucial for understanding functionally important interfaces. Another persistent challenge is the treatment of flexible domains and inter‐domain orientations, which prove difficult in targets such as H1225 and H1220.

Incorporation of ions and small molecules, which are often essential for structural integrity and biological function, also remains suboptimal. Although ion placement was successfully predicted for T1246, even without prior information from the CASP organizers, predictions for other targets, such as T1219, H1202, and H1258, failed to do so. This highlights the need for improved identification and modeling of such molecules as part of the prediction process. Taking the biological context into account, including the protein's cellular location and function, can offer essential guidance. For example, whether a target is extracellular or intracellular influences disulfide bond formation, as illustrated by T1201.

Finally, stoichiometry prediction remains an area for future improvement. Several models failed to correctly capture multimeric assemblies, including more complex cases such as dimer‐of‐dimers or higher‐order assemblies, as seen in T1219.

In summary, CASP16 reflected ongoing progress in protein structure prediction, highlighting the areas where advances have been made. With the observed continued improvements in modeling flexible regions, small molecule interactions, posttranslational modifications, and complex assemblies, structure prediction is becoming a powerful tool for advancing both basic biology and biomedical applications, complementing the experimental work. We are excited to see what progress and challenges the next CASP round will deliver.

## Author Contributions

**Leila T. Alexander:** writing – review and editing, methodology, writing – original draft, project administration. **Océane M. Follonier:** writing – review and editing, writing – original draft, methodology. **Andriy Kryshtafovych:** conceptualization, funding acquisition, methodology, writing – review and editing, project administration. **Kim Abesamis:** investigation, funding acquisition, writing – original draft, methodology, visualization, writing – review and editing, formal analysis, data curation. **Sabrina Bibi‐Triki:** data curation, formal analysis, visualization, writing – review and editing, methodology, investigation, writing – original draft. **Henry G. Box:** investigation, writing – original draft, methodology, visualization, writing – review and editing, formal analysis, data curation. **Cécile Breyton:** investigation, funding acquisition, writing – original draft, methodology, visualization, writing – review and editing, formal analysis, data curation, supervision. **Françoise Bringel:** investigation, writing – original draft, methodology, visualization, writing – review and editing, formal analysis, data curation. **Loic Carrique:** investigation, writing – original draft, methodology, visualization, writing – review and editing, formal analysis, data curation. **Alessio d'Acapito:** investigation, writing – original draft, methodology, visualization, writing – review and editing, formal analysis. **Gang Dong:** investigation, funding acquisition, writing – original draft, methodology, visualization, writing – review and editing, formal analysis, data curation, supervision. **Rebecca DuBois:** investigation, funding acquisition, writing – original draft, methodology, visualization, writing – review and editing, formal analysis, data curation. **Deborah Fass:** investigation, funding acquisition, writing – original draft, methodology, visualization, writing – review and editing, formal analysis, data curation, supervision. **Juliana Martinez Fiesco:** investigation, writing – original draft, funding acquisition, methodology, visualization, writing – review and editing, formal analysis, data curation. **Daniel R. Fox:** investigation, funding acquisition, writing – original draft, methodology, visualization, writing – review and editing, formal analysis, data curation. **Jonathan M. Grimes:** investigation, funding acquisition, writing – original draft, methodology, visualization, writing – review and editing, formal analysis, data curation, supervision. **Rhys Grinter:** investigation, funding acquisition, writing – original draft, methodology, visualization, writing – review and editing, formal analysis, data curation, supervision. **Matthew Jenkins:** investigation, writing – original draft, methodology, visualization, writing – review and editing, formal analysis, data curation. **Roman Kamyshinsky:** investigation, writing – original draft, methodology, visualization, writing – review and editing, formal analysis, data curation. **Jeremy R. Keown:** investigation, funding acquisition, writing – original draft, visualization, methodology, writing – review and editing, formal analysis, data curation, supervision. **Gerald Lackner:** investigation, funding acquisition, writing – original draft, methodology, visualization, writing – review and editing, formal analysis, data curation. **Michael Lammers:** investigation, funding acquisition, writing – original draft, methodology, visualization, writing – review and editing, formal analysis, data curation. **Shiheng Liu:** investigation, funding acquisition, writing – original draft, writing – review and editing, visualization, methodology, formal analysis, data curation, supervision. **Andrew L. Lovering:** investigation, funding acquisition, writing – original draft, writing – review and editing, visualization, methodology, formal analysis, supervision, data curation. **Tomas Malinauskas:** investigation, writing – original draft, methodology, visualization, writing – review and editing, formal analysis, data curation. **Benoît Masquida:** investigation, funding acquisition, writing – original draft, writing – review and editing, visualization, methodology, formal analysis, data curation, supervision. **Gottfried J. Palm:** investigation, funding acquisition, writing – original draft, writing – review and editing, visualization, methodology, formal analysis, data curation. **Christian Siebold:** investigation, funding acquisition, writing – original draft, writing – review and editing, methodology, formal analysis, data curation, supervision. **Tiantian Su:** investigation, funding acquisition, writing – original draft, writing – review and editing, visualization, methodology, formal analysis, data curation, supervision. **Ping Zhang:** investigation, funding acquisition, writing – original draft, writing – review and editing, formal analysis, data curation, supervision, methodology. **Z. Hong Zhou:** investigation, funding acquisition, writing – original draft, visualization, methodology, writing – review and editing, formal analysis, data curation, supervision. **Krzysztof Fidelis:** conceptualization, funding acquisition, writing – review and editing, methodology. **Maya Topf:** conceptualization, funding acquisition, methodology, writing – review and editing. **John Moult:** conceptualization, funding acquisition, writing – review and editing, methodology. **Torsten Schwede:** conceptualization, funding acquisition, writing – review and editing, methodology, supervision.

## Data Availability

Data sharing is not applicable to this article as no data sets were generated or analyzed during the current study.

## References

[1] A. Kryshtafovych , J. Moult , S. G. Bartual , et al., “Target Highlights in CASP9: Experimental Target Structures for the Critical Assessment of Techniques for Protein Structure Prediction,” Proteins 79, no. Suppl 10 (2011): S6–S20, 10.1002/prot.23196. PMC3692002 22020785

[2] A. Kryshtafovych , J. Moult , P. Bales , et al., “Challenging the State of the Art in Protein Structure Prediction: Highlights of Experimental Target Structures for the 10th Critical Assessment of Techniques for Protein Structure Prediction Experiment CASP10,” Proteins 82 (2014): 26–42, 10.1002/prot.24489. 24318984 PMC4072496

[3] A. Kryshtafovych , J. Moult , A. Baslé , et al., “Some of the Most Interesting CASP11 Targets Through the Eyes of Their Authors,” Proteins 84, no. Suppl 1 (2016): S34–S50, 10.1002/prot.24942. PMC4834066 26473983

[4] A. Kryshtafovych , R. Albrecht , A. Baslé , et al., “Target Highlights From the First Post‐PSI CASP Experiment (CASP12, May‐August 2016),” Proteins 86, no. Suppl 1 (2018): S27–S50, 10.1002/prot.25392. PMC5820184 28960539

[5] R. Lepore , A. Kryshtafovych , M. Alahuhta , et al., “Target Highlights in CASP13: Experimental Target Structures Through the Eyes of Their Authors,” Proteins 87, no. 12 (2019): 1037–1057, 10.1002/prot.25805. 31442339 PMC6851490

[6] L. T. Alexander , R. Lepore , A. Kryshtafovych , et al., “Target Highlights in CASP14: Analysis of Models by Structure Providers,” Proteins 89, no. 12 (2021): 1647–1672, 10.1002/prot.26247. 34561912 PMC8616854

[7] L. T. Alexander , J. Durairaj , A. Kryshtafovych , et al., “Protein Target Highlights in CASP15: Analysis of Models by Structure Providers,” Proteins 91, no. 12 (2023): 1571–1599, 10.1002/prot.26545. 37493353 PMC10792529

[8] S. O. Fedechkin , N. L. George , J. T. Wolff , L. M. Kauvar , and R. M. DuBois , “Structures of Respiratory Syncytial Virus G Antigen Bound to Broadly Neutralizing Antibodies,” Science Immunology 3, no. 21 (2018): eaar3534, 10.1126/sciimmunol.aar3534. 29523582 PMC6203301

[9] S. O. Fedechkin , N. L. George , A. M. Nuñez Castrejon , J. R. Dillen , L. M. Kauvar , and R. M. DuBois , “Conformational Flexibility in Respiratory Syncytial Virus G Neutralizing Epitopes,” Journal of Virology 94, no. 6 (2020): e01879‐19, 10.1128/JVI.01879-19. 31852779 PMC7158734

[10] H. G. Jones , T. Ritschel , G. Pascual , et al., “Structural Basis for Recognition of the Central Conserved Region of RSV G by Neutralizing Human Antibodies,” PLoS Pathogens 14, no. 3 (2018): e1006935, 10.1371/journal.ppat.1006935. 29509814 PMC5856423

[11] M. G. Juarez , S. M. O'Rourke , J. V. Dzimianski , et al., “Structures of Respiratory Syncytial Virus G Bound to Broadly Reactive Antibodies Provide Insights Into Vaccine Design,” Scientific Reports 15, no. 1 (2025): 8666, 10.1038/s41598-025-92886-w. 40082629 PMC11906780

[12] M. Ouizougun‐Oubari and R. Fearns , “Structures and Mechanisms of Nonsegmented, Negative‐Strand RNA Virus Polymerases,” Annual Review of Virology 10, no. 1 (2023): 199–215, 10.1146/annurev-virology-111821-102603. 37137281

[13] D. Rubbenstroth , “Avian Bornavirus Research—A Comprehensive Review,” Viruses 14, no. 7 (2022): 1513, 10.3390/v14071513. 35891493 PMC9321243

[14] B. Hoffmann , D. Tappe , D. Höper , et al., “A Variegated Squirrel Bornavirus Associated With Fatal Human Encephalitis,” New England Journal of Medicine 373, no. 2 (2015): 154–162, 10.1056/NEJMoa1415627. 26154788

[15] N. Tarbouriech , F. Chenavier , J. Kawasaki , et al., “Borna Disease Virus 1 Phosphoprotein Forms a Tetramer and Interacts With Host Factors Involved in DNA Double‐Strand Break Repair and mRNA Processing,” Viruses 14, no. 11 (2022): 2358, 10.3390/v14112358. 36366462 PMC9692295

[16] J. D. Whitehead , J. M. Grimes , and J. R. Keown , “Structural and Biophysical Characterization of the Borna Disease Virus 1 Phosphoprotein,” Acta Crystallographica Section F: Structural Biology and Crystallization Communications 79, no. Pt 3 (2023): 51–60, 10.1107/S2053230X23000717. PMC9979977 36862093

[17] A. d'Acapito , A. Decombe , C. A. Arnaud , and C. Breyton , “Comparative Anatomy of Siphophage Tails Before and After Interaction With Their Receptor,” Current Opinion in Structural Biology 92 (2025): 103045, 10.1016/j.sbi.2025.103045. 40279683

[18] R. Linares , C. A. Arnaud , G. Effantin , et al., “Structural Basis of Bacteriophage T5 Infection Trigger and *E. coli* Cell Wall Perforation,” Science Advances 9, no. 12 (2023): eade9674, 10.1126/sciadv.ade9674. 36961893 PMC10038345

[19] S. Degroux , G. Effantin , R. Linares , G. Schoehn , and C. Breyton , “Deciphering Bacteriophage T5 Host Recognition Mechanism and Infection Trigger,” Journal of Virology 97, no. 3 (2023): e0158422, 10.1128/jvi.01584-22. 36779755 PMC10062170

[20] C. Garcia‐Doval , J. R. Castón , D. Luque , et al., “Structure of the Receptor‐Binding Carboxy‐Terminal Domain of the Bacteriophage T5 L‐Shaped Tail Fibre With and Without Its Intra‐Molecular Chaperone,” Viruses 7, no. 12 (2015): 6424–6440, 10.3390/v7122946. 26670244 PMC4690869

[21] J. Jumper , R. Evans , A. Pritzel , et al., “Highly Accurate Protein Structure Prediction With AlphaFold,” Nature 596, no. 7873 (2021): 583–589, 10.1038/s41586-021-03819-2. 34265844 PMC8371605

[22] B. Maucourt , S. Vuilleumier , and F. Bringel , “Transcriptional Regulation of Organohalide Pollutant Utilisation in Bacteria,” FEMS Microbiology Reviews 44, no. 2 (2020): 189–207, 10.1093/femsre/fuaa002. 32011697

[23] E. E. L. Muller , E. Hourcade , Y. Louhichi‐Jelail , P. Hammann , S. Vuilleumier , and F. Bringel , “Functional Genomics of Dichloromethane Utilization in *Methylobacterium extorquens* DM4,” Environmental Microbiology 13, no. 9 (2011): 2518–2535, 10.1111/j.1462-2920.2011.02524.x. 21854516

[24] S. Bibi‐Triki , G. Husson , B. Maucourt , S. Vuilleumier , C. Carapito , and F. Bringel , “N‐Terminome and Proteogenomic Analysis of the *Methylobacterium extorquens* DM4 Reference Strain for Dichloromethane Utilization,” Journal of Proteomics 179 (2018): 131–139, 10.1016/j.jprot.2018.03.012. 29567292

[25] L. Holm , “Dali Server: Structural Unification of Protein Families,” Nucleic Acids Research 50, no. W1 (2022): W210–W215, 10.1093/nar/gkac387. 35610055 PMC9252788

[26] S. W. Cowan , M. E. Newcomer , and T. A. Jones , “Crystallographic Studies on a Family of Cellular Lipophilic Transport Proteins. Refinement of P2 Myelin Protein and the Structure Determination and Refinement of Cellular Retinol‐Binding Protein in Complex With All‐Trans‐Retinol,” Journal of Molecular Biology 230, no. 4 (1993): 1225–1246, 10.1006/jmbi.1993.1238. 7683727

[27] V. Calderone , R. Berni , and G. Zanotti , “High‐Resolution Structures of Retinol‐Binding Protein in Complex With Retinol: pH‐Induced Protein Structural Changes in the Crystal State,” Journal of Molecular Biology 329, no. 4 (2003): 841–850, 10.1016/s0022-2836(03)00468-6. 12787682

[28] E. Jakobsson , G. Alvite , T. Bergfors , A. Esteves , and G. J. Kleywegt , “The Crystal Structure of Echinococcus Granulosus Fatty‐Acid‐Binding Protein 1,” Biochimica et Biophysica Acta 1649, no. 1 (2003): 40–50, 10.1016/s1570-9639(03)00151-1. 12818189

[29] S. Capaldi , M. Guariento , G. Saccomani , D. Fessas , M. Perduca , and H. L. Monaco , “A Single Amino Acid Mutation in Zebrafish ( *Danio rerio* ) Liver Bile Acid‐Binding Protein Can Change the Stoichiometry of Ligand Binding,” Journal of Biological Chemistry 282, no. 42 (2007): 31008–31018, 10.1074/jbc.M705399200. 17670743

[30] R. E. Gillilan , S. D. Ayers , and N. Noy , “Structural Basis for Activation of Fatty Acid‐Binding Protein 4,” Journal of Molecular Biology 372, no. 5 (2007): 1246–1260, 10.1016/j.jmb.2007.07.040. 17761196 PMC2032018

[31] C. M. Bianchetti , G. C. Blouin , E. Bitto , J. S. Olson , and G. N. Phillips , “The Structure and NO Binding Properties of the Nitrophorin‐Like Heme‐Binding Protein From *Arabidopsis thaliana* Gene Locus At1g79260.1,” Proteins 78, no. 4 (2010): 917–931, 10.1002/prot.22617. 19938152 PMC2811769

[32] G. Bashiri , C. J. Squire , N. J. Moreland , and E. N. Baker , “Crystal Structures of F420‐Dependent Glucose‐6‐Phosphate Dehydrogenase FGD1 Involved in the Activation of the Anti‐Tuberculosis Drug Candidate PA‐824 Reveal the Basis of Coenzyme and Substrate Binding,” Journal of Biological Chemistry 283, no. 25 (2008): 17531–17541, 10.1074/jbc.M801854200. 18434308

[33] J. S. Richardson , L. L. Videau , C. J. Williams , B. J. Hintze , S. M. Lewis , and D. C. Richardson , “ *Cis*‐nonProline Peptides: Genuine Occurrences and Their Functional Roles,” bioRxiv, 2018, 10.1101/324517. PMC12102755 40411433

[34] R. E. Sockett , “Predatory Lifestyle of *Bdellovibrio bacteriovorus* ,” Annual Review of Microbiology 63 (2009): 523–539, 10.1146/annurev.micro.091208.073346. 19575566

[35] S. Rendulic , P. Jagtap , A. Rosinus , et al., “A Predator Unmasked: Life Cycle of *Bdellovibrio bacteriovorus* From a Genomic Perspective,” Science 303, no. 5658 (2004): 689–692, 10.1126/science.1093027. 14752164

[36] C. Lambert , C. Y. Chang , M. J. Capeness , and R. E. Sockett , “The First Bite—Profiling the Predatosome in the Bacterial Pathogen Bdellovibrio,” PLoS One 5, no. 1 (2010): e8599, 10.1371/journal.pone.0008599. 20062540 PMC2797640

[37] D. Gadkari and H. Stolp , “Energy Metabolism of *Bdellovibrio bacteriovorus*. I. Energy Production, ATP Pool, Energy Charge,” Archives of Microbiology 102, no. 3 (1975): 179–185, 10.1007/BF00428366. 1156083

[38] L. Hobley , R. K. Y. Fung , C. Lambert , et al., “Discrete Cyclic Di‐GMP‐Dependent Control of Bacterial Predation Versus Axenic Growth in *Bdellovibrio bacteriovorus* ,” PLoS Pathogens 8, no. 2 (2012): e1002493, 10.1371/journal.ppat.1002493. 22319440 PMC3271064

[39] R. C. Molina‐Quiroz , C. Silva‐Valenzuela , J. Brewster , E. Castro‐Nallar , S. B. Levy , and A. Camilli , “Cyclic AMP Regulates Bacterial Persistence Through Repression of the Oxidative Stress Response and SOS‐Dependent DNA Repair in Uropathogenic *Escherichia coli* ,” MBio 9, no. 1 (2018): e02144‐17, 10.1128/mBio.02144-17. 29317513 PMC5760743

[40] J. Green , M. R. Stapleton , L. J. Smith , et al., “Cyclic‐AMP and Bacterial Cyclic‐AMP Receptor Proteins Revisited: Adaptation for Different Ecological Niches,” Current Opinion in Microbiology 18, no. 100 (2014): 1–7, 10.1016/j.mib.2014.01.003. 24509484 PMC4005916

[41] S. H. Seok , H. Im , H. S. Won , et al., “Structures of Inactive CRP Species Reveal the Atomic Details of the Allosteric Transition That Discriminates Cyclic Nucleotide Second Messengers,” Acta Crystallographica, Section D: Biological Crystallography 70, no. Pt 6 (2014): 1726–1742, 10.1107/S139900471400724X. 24914983

[42] I. T. Cadby , S. M. Basford , R. Nottingham , et al., “Nucleotide Signaling Pathway Convergence in a cAMP‐Sensing Bacterial c‐di‐GMP Phosphodiesterase,” EMBO Journal 38, no. 17 (2019): e100772, 10.15252/embj.2018100772. 31355487 PMC6717892

[43] L. Hazlett and M. Wu , “Defensins in Innate Immunity,” Cell and Tissue Research 343, no. 1 (2011): 175–188, 10.1007/s00441-010-1022-4. 20730446

[44] C. L. Bevins and N. H. Salzman , “Paneth Cells, Antimicrobial Peptides and Maintenance of Intestinal Homeostasis,” Nature Reviews. Microbiology 9, no. 5 (2011): 356–368, 10.1038/nrmicro2546. 21423246

[45] A. Szyk , Z. Wu , K. Tucker , D. Yang , W. Lu , and J. Lubkowski , “Crystal Structures of Human Alpha‐Defensins HNP4, HD5, and HD6,” Protein Science 15, no. 12 (2006): 2749–2760, 10.1110/ps.062336606. 17088326 PMC2242434

[46] P. Chairatana and E. M. Nolan , “Molecular Basis for Self‐Assembly of a Human Host‐Defense Peptide That Entraps Bacterial Pathogens,” Journal of the American Chemical Society 136, no. 38 (2014): 13267–13276, 10.1021/ja5057906. 25158166 PMC4183631

[47] E. G. Healey , B. Bishop , J. Elegheert , C. H. Bell , S. Padilla‐Parra , and C. Siebold , “Repulsive Guidance Molecule Is a Structural Bridge Between Neogenin and Bone Morphogenetic Protein,” Nature Structural & Molecular Biology 22, no. 6 (2015): 458–465, 10.1038/nsmb.3016. PMC4456160 25938661

[48] T. Malinauskas , T. V. Peer , B. Bishop , T. D. Mueller , and C. Siebold , “Repulsive Guidance Molecules Lock Growth Differentiation Factor 5 in an Inhibitory Complex,” Proceedings of the National Academy of Sciences of the United States of America 117, no. 27 (2020): 15620–15631, 10.1073/pnas.2000561117. 32576689 PMC7354924

[49] T. Malinauskas , G. Moore , A. F. Rudolf , et al., “Molecular Mechanism of BMP Signal Control by Twisted Gastrulation,” Nature Communications 15, no. 1 (2024): 4976, 10.1038/s41467-024-49065-8. PMC11167000 38862520

[50] J. Söding , A. Biegert , and A. N. Lupas , “The HHpred Interactive Server for Protein Homology Detection and Structure Prediction,” Nucleic Acids Research 33, no. Web Server issue (2005): W244–W248, 10.1093/nar/gki408. 15980461 PMC1160169

[51] H. Schreuder , A. Liesum , J. Pohl , M. Kruse , and M. Koyama , “Crystal Structure of Recombinant Human Growth and Differentiation Factor 5: Evidence for Interaction of the Type I and Type II Receptor‐Binding Sites,” Biochemical and Biophysical Research Communications 329, no. 3 (2005): 1076–1086, 10.1016/j.bbrc.2005.02.078. 15752764

[52] A. Kotzsch , J. Nickel , A. Seher , W. Sebald , and T. D. Müller , “Crystal Structure Analysis Reveals a Spring‐Loaded Latch as Molecular Mechanism for GDF‐5‐Type I Receptor Specificity,” EMBO Journal 28, no. 7 (2009): 937–947, 10.1038/emboj.2009.37. 19229295 PMC2670865

[53] U. Klammert , T. D. Mueller , T. V. Hellmann , et al., “GDF‐5 Can Act as a Context‐Dependent BMP‐2 Antagonist,” BMC Biology 13 (2015): 77, 10.1186/s12915-015-0183-8. 26385096 PMC4575486

[54] K. Nolan , C. Kattamuri , S. A. Rankin , R. J. Read , A. M. Zorn , and T. B. Thompson , “Structure of Gremlin‐2 in Complex With GDF5 Gives Insight Into DAN‐Family‐Mediated BMP Antagonism,” Cell Reports 16, no. 8 (2016): 2077–2086, 10.1016/j.celrep.2016.07.046. 27524626 PMC5001929

[55] D. Akinbosede , R. Chizea , and S. A. Hare , “Pirates of the Haemoglobin,” Microbial Cell 9, no. 4 (2022): 84–102, 10.15698/mic2022.04.775. 35434122 PMC8977872

[56] D. R. Fox , K. Asadollahi , I. Samuels , et al., “Inhibiting Heme‐Piracy by Pathogenic *Escherichia coli* Using *De Novo*‐Designed Proteins,” bioRxiv, 2024, 10.1101/2024.12.05.626953. PMC12241658 40634285

[57] D. R. Fox , I. Samuels , S. Binks , and R. Grinter , “The Structure of a Haemoglobin‐Nanobody Complex Reveals Human β‐Subunit‐Specific Interactions,” FEBS Letters 598, no. 18 (2024): 2240–2248, 10.1002/1873-3468.14958. 38880764

[58] T. Delfin‐Riela , M. A. Rossotti , C. Echaides , and G. González‐Sapienza , “A Nanobody‐Based Test for Highly Sensitive Detection of Hemoglobin in Fecal Samples,” Analytical and Bioanalytical Chemistry 412, no. 2 (2020): 389–396, 10.1007/s00216-019-02246-7. 31760451

[59] S. Muyldermans , “Nanobodies: Natural Single‐Domain Antibodies,” Annual Review of Biochemistry 82 (2013): 775–797, 10.1146/annurev-biochem-063011-092449. 23495938

[60] J. Abramson , J. Adler , J. Dunger , et al., “Accurate Structure Prediction of Biomolecular Interactions With AlphaFold 3,” Nature 630, no. 8016 (2024): 493–500, 10.1038/s41586-024-07487-w. 38718835 PMC11168924

[61] Chai Discovery , J. Boitreaud , J. Dent , et al., “Chai‐1: Decoding the Molecular Interactions of Life,” bioRxiv, 2024, 10.1101/2024.10.10.615955.

[62] H. N. Eisen , “Affinity Enhancement of Antibodies: How Low‐Affinity Antibodies Produced Early in Immune Responses Are Followed by High‐Affinity Antibodies Later and in Memory B‐Cell Responses,” Cancer Immunology Research 2, no. 5 (2014): 381–392, 10.1158/2326-6066.CIR-14-0029. 24795350

[63] R. Yin and B. G. Pierce , “Evaluation of AlphaFold Antibody‐Antigen Modeling With Implications for Improving Predictive Accuracy,” Protein Science 33, no. 1 (2024): e4865, 10.1002/pro.4865. 38073135 PMC10751731

[64] M. F. Lensink , G. Brysbaert , N. Raouraoua , et al., “Impact of AlphaFold on Structure Prediction of Protein Complexes: The CASP15‐CAPRI Experiment,” Proteins 91, no. 12 (2023): 1658–1683, 10.1002/prot.26609. 37905971 PMC10841881

[65] F. N. Hitawala and J. J. Gray , “What Does AlphaFold3 Learn About Antigen and Nanobody Docking, and What Remains Unsolved?,” bioRxiv, 2024, 10.1101/2024.09.21.614257. PMC12360200 40814020

[66] A. Harmalkar , S. Lyskov , and J. J. Gray , “Reliable Protein‐Protein Docking With AlphaFold, Rosetta, and Replica‐Exchange,” bioRxiv, 2025, 10.1101/2023.07.28.551063. PMC12113263 40424178

[67] N. R. Bennett , J. L. Watson , R. J. Ragotte , et al., “Atomically Accurate De Novo Design of Antibodies With RFdiffusion,” bioRxiv, 2024, 10.1101/2024.03.14.585103. PMC12727541 41193805

[68] K. Gull , “The Cytoskeleton of Trypanosomatid Parasites,” Annual Review of Microbiology 53 (1999): 629–655, 10.1146/annurev.micro.53.1.629. 10547703

[69] M. C. Field and M. Carrington , “The Trypanosome Flagellar Pocket,” Nature Reviews. Microbiology 7, no. 11 (2009): 775–786, 10.1038/nrmicro2221. 19806154

[70] S. Lacomble , S. Vaughan , C. Gadelha , et al., “Three‐Dimensional Cellular Architecture of the Flagellar Pocket and Associated Cytoskeleton in Trypanosomes Revealed by Electron Microscope Tomography,” Journal of Cell Science 122, no. Pt 8 (2009): 1081–1090, 10.1242/jcs.045740. 19299460 PMC2714436

[71] M. Bonhivers , S. Nowacki , N. Landrein , and D. R. Robinson , “Biogenesis of the Trypanosome Endo‐Exocytotic Organelle Is Cytoskeleton Mediated,” PLoS Biology 6, no. 5 (2008): e105, 10.1371/journal.pbio.0060105. 18462016 PMC2365980

[72] C. Florimond , A. Sahin , K. Vidilaseris , et al., “BILBO1 Is a Scaffold Protein of the Flagellar Pocket Collar in the Pathogen *Trypanosoma brucei* ,” PLoS Pathogens 11, no. 3 (2015): e1004654, 10.1371/journal.ppat.1004654. 25822645 PMC4379179

[73] K. Vidilaseris , B. Morriswood , G. Kontaxis , and G. Dong , “Structure of the TbBILBO1 Protein N‐Terminal Domain From *Trypanosoma brucei* Reveals an Essential Requirement for a Conserved Surface Patch,” Journal of Biological Chemistry 289, no. 6 (2014): 3724–3735, 10.1074/jbc.M113.529032. 24362019 PMC3916570

[74] K. Vidilaseris , E. Shimanovskaya , H. J. Esson , B. Morriswood , and G. Dong , “Assembly Mechanism of *Trypanosoma brucei* BILBO1, a Multidomain Cytoskeletal Protein,” Journal of Biological Chemistry 289, no. 34 (2014): 23870–23881, 10.1074/jbc.M114.554659. 25031322 PMC4156054

[75] K. Vidilaseris , J. Lesigang , B. Morriswood , and G. Dong , “Assembly Mechanism of *Trypanosoma brucei* BILBO1 at the Flagellar Pocket Collar,” Communicative & Integrative Biology 8, no. 1 (2015): e992739, 10.4161/19420889.2014.992739. 26844754 PMC4594465

[76] C. E. Broster Reix , M. R. Ramanantsalama , C. Di Primo , et al., “Intrabody‐Induced Cell Death by Targeting the *T. brucei* Cytoskeletal Protein TbBILBO1,” Microbiology Spectrum 9, no. 2 (2021): e0091521, 10.1128/Spectrum.00915-21. 34704826 PMC8549753

[77] D. A. Roosen and M. R. Cookson , “LRRK2 at the Interface of Autophagosomes, Endosomes and Lysosomes,” Molecular Neurodegeneration 11, no. 1 (2016): 73, 10.1186/s13024-016-0140-1. 27927216 PMC5142374

[78] M. Madureira , N. Connor‐Robson , and R. Wade‐Martins , “LRRK2: Autophagy and Lysosomal Activity,” Frontiers in Neuroscience 14 (2020): 498, 10.3389/fnins.2020.00498. 32523507 PMC7262160

[79] R. Watanabe , R. Buschauer , J. Böhning , et al., “The In Situ Structure of Parkinson's Disease‐Linked LRRK2,” Cell 182, no. 6 (2020): 1508–1518.e16, 10.1016/j.cell.2020.08.004. 32783917 PMC7869717

[80] A. Myasnikov , H. Zhu , P. Hixson , et al., “Structural Analysis of the Full‐Length Human LRRK2,” Cell 184, no. 13 (2021): 3519–3527.e10, 10.1016/j.cell.2021.05.004. 34107286 PMC8887629

[81] D. M. Snead , M. Matyszewski , A. M. Dickey , Y. X. Lin , A. E. Leschziner , and S. L. Reck‐Peterson , “Structural Basis for Parkinson's Disease‐Linked LRRK2's Binding to Microtubules,” Nature Structural & Molecular Biology 29, no. 12 (2022): 1196–1207, 10.1038/s41594-022-00863-y. PMC9758056 36510024

[82] T. Gasser , “Molecular Pathogenesis of Parkinson Disease: Insights From Genetic Studies,” Expert Reviews in Molecular Medicine 11 (2009): e22, 10.1017/S1462399409001148. 19631006

[83] W. Satake , Y. Nakabayashi , I. Mizuta , et al., “Genome‐Wide Association Study Identifies Common Variants at Four Loci as Genetic Risk Factors for Parkinson's Disease,” Nature Genetics 41, no. 12 (2009): 1303–1307, 10.1038/ng.485. 19915576

[84] J. Simón‐Sánchez , C. Schulte , J. M. Bras , et al., “Genome‐Wide Association Study Reveals Genetic Risk Underlying Parkinson's Disease,” Nature Genetics 41, no. 12 (2009): 1308–1312, 10.1038/ng.487. 19915575 PMC2787725

[85] C. M. Lill , J. T. Roehr , M. B. McQueen , et al., “Comprehensive Research Synopsis and Systematic Meta‐Analyses in Parkinson's Disease Genetics: The PDGene Database,” PLoS Genetics 8, no. 3 (2012): e1002548, 10.1371/journal.pgen.1002548. 22438815 PMC3305333

[86] T. Eguchi , T. Kuwahara , M. Sakurai , et al., “LRRK2 and Its Substrate Rab GTPases Are Sequentially Targeted Onto Stressed Lysosomes and Maintain Their Homeostasis,” Proceedings of the National Academy of Sciences of the United States of America 115, no. 39 (2018): E9115–E9124, 10.1073/pnas.1812196115. 30209220 PMC6166828

[87] E. Purlyte , H. S. Dhekne , A. R. Sarhan , et al., “Rab29 Activation of the Parkinson's Disease‐Associated LRRK 2 Kinase,” EMBO Journal 38, no. 2 (2019): e101237, 10.15252/embj.2018101237. 30647193 PMC6331717

[88] X. Wang , V. V. Bondar , O. B. Davis , et al., “Rab12 Is a Regulator of LRRK2 and Its Activation by Damaged Lysosomes,” eLife 12 (2023): e87255, 10.7554/eLife.87255. 37874617 PMC10708889

[89] N. Dzamko , M. Deak , F. Hentati , et al., “Inhibition of LRRK2 Kinase Activity Leads to Dephosphorylation of Ser910/Ser935, Disruption of 14‐3‐3 Binding and Altered Cytoplasmic Localization,” Biochemical Journal 430, no. 3 (2010): 405–413, 10.1042/BJ20100784. 20659021 PMC3631100

[90] J. Zhao , T. P. Molitor , J. W. Langston , and R. J. Nichols , “LRRK2 Dephosphorylation Increases Its Ubiquitination,” Biochemical Journal 469, no. 1 (2015): 107–120, 10.1042/BJ20141305. 25939886 PMC4613513

[91] R. J. Nichols , N. Dzamko , N. A. Morrice , et al., “14‐3‐3 Binding to LRRK2 Is Disrupted by Multiple Parkinson's Disease‐Associated Mutations and Regulates Cytoplasmic Localization,” Biochemical Journal 430, no. 3 (2010): 393–404, 10.1042/BJ20100483. 20642453 PMC2932554

[92] X. Li , Q. J. Wang , N. Pan , et al., “Phosphorylation‐Dependent 14‐3‐3 Binding to LRRK2 Is Impaired by Common Mutations of Familial Parkinson's Disease,” PLoS One 6, no. 3 (2011): e17153, 10.1371/journal.pone.0017153. 21390248 PMC3046972

[93] K. Muda , D. Bertinetti , F. Gesellchen , et al., “Parkinson‐Related LRRK2 Mutation R1441C/G/H Impairs PKA Phosphorylation of LRRK2 and Disrupts Its Interaction With 14‐3‐3,” Proceedings of the National Academy of Sciences of the United States of America 111, no. 1 (2014): E34–E43, 10.1073/pnas.1312701111. 24351927 PMC3890784

[94] D. H. Jones , S. Ley , and A. Aitken , “Isoforms of 14‐3‐3 Protein Can Form Homo‐ and Heterodimers In Vivo and In Vitro: Implications for Function as Adapter Proteins,” FEBS Letters 368, no. 1 (1995): 55–58, 10.1016/0014-5793(95)00598-4. 7615088

[95] M. Chaudhri , M. Scarabel , and A. Aitken , “Mammalian and Yeast 14‐3‐3 Isoforms Form Distinct Patterns of Dimers In Vivo,” Biochemical and Biophysical Research Communications 300, no. 3 (2003): 679–685, 10.1016/S0006-291X(02)02902-9. 12507503

[96] X. Yang , W. H. Lee , F. Sobott , et al., “Structural Basis for Protein–Protein Interactions in the 14‐3‐3 Protein Family,” Proceedings of the National Academy of Sciences of the United States of America 103, no. 46 (2006): 17237–17242, 10.1073/pnas.0605779103. 17085597 PMC1859916

[97] E. P. Hoffman , R. H. Brown , and L. M. Kunkel , “Dystrophin: The Protein Product of the Duchenne Muscular Dystrophy Locus,” Cell 51, no. 6 (1987): 919–928, 10.1016/0092-8674(87)90579-4. 3319190

[98] E. Mercuri , C. G. Bönnemann , and F. Muntoni , “Muscular Dystrophies,” Lancet 394, no. 10213 (2019): 2025–2038, 10.1016/S0140-6736(19)32910-1. 31789220

[99] M. Koenig , E. P. Hoffman , C. J. Bertelson , A. P. Monaco , C. Feener , and L. M. Kunkel , “Complete Cloning of the Duchenne Muscular Dystrophy (DMD) cDNA and Preliminary Genomic Organization of the DMD Gene in Normal and Affected Individuals,” Cell 50, no. 3 (1987): 509–517, 10.1016/0092-8674(87)90504-6. 3607877

[100] K. P. Campbell and S. D. Kahl , “Association of Dystrophin and an Integral Membrane Glycoprotein,” Nature 338, no. 6212 (1989): 259–262, 10.1038/338259a0. 2493582

[101] M. Yoshida and E. Ozawa , “Glycoprotein Complex Anchoring Dystrophin to Sarcolemma,” Journal of Biochemistry 108, no. 5 (1990): 748–752, 10.1093/oxfordjournals.jbchem.a123276. 2081733

[102] J. M. Ervasti and K. P. Campbell , “Membrane Organization of the Dystrophin‐Glycoprotein Complex,” Cell 66, no. 6 (1991): 1121–1131, 10.1016/0092-8674(91)90035-w. 1913804

[103] O. Ibraghimov‐Beskrovnaya , J. M. Ervasti , C. J. Leveille , C. A. Slaughter , S. W. Sernett , and K. P. Campbell , “Primary Structure of Dystrophin‐Associated Glycoproteins Linking Dystrophin to the Extracellular Matrix,” Nature 355, no. 6362 (1992): 696–702, 10.1038/355696a0. 1741056

[104] S. Liu , T. Su , X. Xia , and Z. H. Zhou , “Native DGC Structure Rationalizes Muscular Dystrophy‐Causing Mutations,” Nature 637, no. 8048 (2025): 1261–1271, 10.1038/s41586-024-08324-w. 39663457 PMC11936492

